# Functional Genomic Analysis of the Impact of Camelina (*Camelina sativa*) Meal on Atlantic Salmon (*Salmo salar*) Distal Intestine Gene Expression and Physiology

**DOI:** 10.1007/s10126-016-9704-x

**Published:** 2016-06-02

**Authors:** Tyler D. Brown, Tiago S. Hori, Xi Xue, Chang Lin Ye, Derek M. Anderson, Matthew L. Rise

**Affiliations:** Department of Ocean Sciences, Memorial University of Newfoundland, 1 Marine Lab Road, St. John’s, NL A1C 5S7 Canada; Department of Plant and Animal Sciences, Faculty of Agriculture, Dalhousie University, Truro, NS Canada B2N 5E3

**Keywords:** Camelina meal, Atlantic salmon, Inflammation, Functional genomics, Distal intestine

## Abstract

The inclusion of plant meals in diets of farmed Atlantic salmon can elicit inflammatory responses in the distal intestine (DI). For the present work, fish were fed a standard fish meal (FM) diet or a diet with partial replacement of FM with solvent-extracted camelina meal (CM) (8, 16, or 24 % CM inclusion) during a 16-week feeding trial. A significant decrease in growth performance was seen in fish fed all CM inclusion diets (Hixson et al. in Aquacult Nutr 22:615–630, 2016). A 4x44K oligonucleotide microarray experiment was carried out and significance analysis of microarrays (SAM) and rank products (RP) methods were used to identify differentially expressed genes between the DIs of fish fed the 24 % CM diet and those fed the FM diet. Twelve features representing six known transcripts and two unknowns were identified as CM responsive by both SAM and RP. The six known transcripts (including *thioredoxin* and *ependymin*), in addition to *tgfb*, *mmp13*, and *GILT*, were studied using qPCR with RNA templates from all four experimental diet groups. All six microarray-identified genes were confirmed to be CM responsive, as was *tgfb* and *mmp13*. Histopathological analyses identified signs of inflammation in the DI of salmon fed CM-containing diets, including lamina propria and sub-epithelial mucosa thickening, infiltration of eosinophilic granule cells, increased goblet cells and decreased enterocyte vacuolization. All of these were significantly altered in 24 % CM compared to all other diets, with the latter two also altered in 16 % CM compared with 8 % CM and control diet groups. Significant correlation was seen between histological parameters as well as between five of the qPCR analyzed genes and histological parameters. These molecular biomarkers of inflammation arising from long-term dietary CM exposure will be useful in the development of CM-containing diets that do not have deleterious effects on salmon growth or physiology.

**Electronic supplementary material:**

The online version of this article (doi:10.1007/s10126-016-9704-x) contains supplementary material, which is available to authorized users.

## Introduction

Fish products are an important component of many human diets, and the demand for marine products is increasing as the global population has grown past seven billion (FAO 2009). The increasing demand for fish products for human consumption has fueled a rapid increase in the production of the aquaculture industry and its continued growth is required to meet the global demand. In 2003, the global production from aquaculture of aquatic animals was approximately 39 million tonnes, while in 2012, this production had reached approximately 67 million tonnes; in this same time, the global production from capture fisheries remained relatively unchanged, at around 90 million tonnes per year (FAO 2012).

Atlantic salmon (*Salmo salar*) is a carnivorous species and, as such, aquaculture feeds for farmed Atlantic salmon typically include marine animal-based protein and oil sources (such as fish and crustacean) that rely heavily on the inputs of unsustainable fishery resources (Tacon et al. 2009). The supplies of these raw marine products are limited and include fish oil (FO) and fish meal (FM) from natural stocks of lower trophic level fish species such as anchovy and herring (Tacon et al. 2009). The global output of FO and FM is restricted as the populations of some natural fish stocks are declining due to over-exploitation (Tacon and Metian 2009). Therefore, sustainable, cost-effective substitutes for FM are currently being sought with many plant-based alternatives being investigated (Tacon and Metian 2008). Plant-based products are not typically part of the diet of wild Atlantic salmon, and the supplementation of feeds for Atlantic salmon with plant meals (PM) may lead to inflammation of the distal intestine (DI) (enteritis) (Chikwati et al. 2013; Marjara et al. 2012; Moldal et al. 2014; Overland et al. 2009; Sahlmann et al. 2013). This inflammation is typically characterized by the shortening of intestinal villi, a decrease in supranuclear vacuoles (SNV) in the intestinal epithelial cells, significant infiltration of inflammatory cells in the lamina propria (LP), an increase in the number of goblet cells (GC), and thickening of the sub-epithelial mucosa (SEM) (Baeverfjord and Krogdahl 1996). The presence of inflammation is preceded by changes in the expression of genes that are responsible for mediating inflammatory responses. This inflammatory response, paired with many other factors, leads to decreased digestibility of essential nutrients and a decrease in the overall growth performance of the fish (Chikwati et al. 2013; Marjara et al. 2012; Moldal et al. 2014; Overland et al. 2009; Sahlmann et al. 2013). The specific components of PMs that are responsible for this inflammatory response are not known for certain. However, many plant products contain antinutritional factors (Sahlmann et al. 2013), and these components may be responsible for the inflammatory response seen in the intestine. In camelina, the main antinutrients are glucosinolates, phytic acid, sinapine, and tannins (Russo and Reggiani 2012). Glucosinolates and their metabolites are known to cause impairment of growth and irritation of the gastrointestinal tract. Phytic acid and tannins have been shown to render essential minerals insoluble and inhibit digestive enzymes, respectively, which may also inhibit growth due to decreased utilization of vitamins and minerals (Amarowicz et al. 2010; Schlemmer et al. 2009). In Atlantic salmon, the hindgut is the main site of amino acid absorption in the digestive tract and, as such, any loss of function that may be caused by inflammation can lead to a decline in the growth performance of the fish (Lokka et al. 2013; Sire et al. 1981).

A novel source of plant-based protein that is currently being considered as a supplementation to FM is the meal from camelina (*Camelina sativa*). Camelina is a flowering oilseed crop of the Brassicaceae family with many characteristics that are favorable to agriculture. These include low fertilizer and nitrogen requirements and high resistance to biotic and abiotic stress (Ghamkhar et al. 2010). CM contains up to 45 % crude protein and is comparable in this regard to other oilseed crops such as canola and rapeseed (Acamovic et al. 1999; Frame et al. 2007; Hixson et al. 2016). CM also contains adequate levels of at least 18 amino acids, with nine of them being essential, and only requires supplementation of some limiting amino acids such as methionine and/or lysine (Cherian 2012; Zubr 2003).

Recently, a feeding trial was conducted to examine the growth performance of Atlantic salmon-fed diets with partial replacement of FM with CM. The current study uses some of the same diets in the same feeding trial as Hixson et al. (2016), which focused on tissue lipid and amino acid analyses. The current study examines the effect of CM-supplemented diets on DI transcript expression and histology. Functional genomics analyses of hindgut tissue samples were carried out using DNA microarrays and real-time quantitative polymerase chain reaction (qPCR). These techniques have been used in previous studies for nutrigenomic analyses to assess the impact of other PM inclusion on the hindgut of Atlantic salmon (Chikwati et al. 2013; Marjara et al. 2012; Moldal et al. 2014; Overland et al. 2009; Sahlmann et al. 2013). The current functional genomics study aimed to identify and validate putative inflammatory biomarker genes that are responsive to long-term exposure to CM-containing aquafeeds. The gene expression data were paired with histological data of the hindgut tissue to allow for a more thorough understanding of the effect of CM on the Atlantic salmon hindgut. Molecular biomarkers of chronic inflammation arising from this study will be useful in future research aimed at determining the maximum inclusion of CM that does not cause deleterious effects in the Atlantic salmon DI.

A study (Ye 2014) analyzing the effect of high oil residue CM, rather than solvent-extracted CM (SECM, which was used in the current study), on growth performance, carcass composition, and DI histology of Atlantic salmon smolts was recently carried out; in addition to using a different CM product, Ye (2014) is different from the current study in that it did not include gene expression assays, and the two studies used distinct methods for histological analyses. Another recent study (Ye et al. 2016) evaluated the effects of SECM-containing diets on Atlantic salmon parr using carcass composition analysis and the same semi-quantitative histological methods as in Ye (2014).

## Materials and Methods

### Diet Formulation

Camelina (Calena cultivar) was grown and harvested by the staff of the Dalhousie Agricultural Campus in Canning, NS, Canada. The seeds were cold pressed using a KEK 0500 screw press at Atlantic Oilseed Processing (Summerside, PEI, Canada). The resulting meal was ground with a hammer mill (screen size 8 mm) into a pre-pressed meal cake at Atlantic Oilseed Processing, and solvent extracted with petroleum ether at a concentration of 3 mL g^−1^ at the Faculty of Agriculture Campus, Dalhousie University (Truro, NS, Canada). The solvent-extracted CM used in this study contained 39 % crude protein and had a gross energy content of 4,320 kcal/kg (Hixson et al. 2016; Ye 2014).

All diets in this trial were formulated at the Dalhousie Agricultural Campus to be iso-nitrogenous, iso-energetic practical diets and were formulated to meet the nutritional requirements of Atlantic salmon (National Research Council 2011). The experimental diets included a control diet with 34.9 % FM; and CM-containing diets with partial replacement of FM with 8 % CM (8CM); 16 % CM (16CM), or 24 % CM (24CM). The diet formulations are outlined in Table 1.

**Table 1:** Formulation and composition of control and experimental diets

Ingredients	0CM	8CM	16CM	24CM
Wheat gluten meal	15	15	15	15
Empyreal 75®	5.0	5.0	5.0	5.0
D/L methionine	0.17	0.17	0.17	0.17
Vitamin mineral premix^a^	0.2	0.2	0.2	0.2
Antioxidant/pigment premix	0.25	0.25	0.25	0.25
Choline chloride	0.5	0.5	0.5	0.5
Whey	5.0	5.0	5.0	5.0
Pregelatinized starch	2.5	2.5	2.5	2.5
Fish meal	34.9	34.2	29.9	27.4
Fish oil	14.0	15.7	17.3	18.9
Wheat middlings	22.4	15.3	8.2	1.0
Camelina meal	0	8.0	16	24

Diet formulations were previously reported in Hixson et al. (2016)

Values presented as % *w*/*w* of total feed composition

^a^Vitamin/mineral premix contains per kilogram—77.5 mg zinc, 125 mg manganese, 84 mg iron, 2.5 mg copper, 7.5 mg iodine, 5,000 IU vitamin A, 4000 IU vitamin D, 2 mg vitamin K, 4 mg vitamin B12, 8 mg thiamine, 18 mg riboflavin, 40 mg pantothenic acid, 100 mg niacin, 4 mg folic acid, 0.6 mg biotin, 15 mg pyridoxine, 100 mg inositol, 42 mg ethoxyquin, 1372 mg wheat shorts

### Experimental Animals

A feeding trial experiment was conducted in accordance with the regulations set out by the Canadian Council of Animal Care for the ethical treatment of animals (Memorial University animal care protocol: 12-50-MR). This feeding trial was carried out in the Dr. Joe Brown Aquatic Research Building (JBARB) at the Ocean Sciences Centre (OSC) of Memorial University of Newfoundland (Canada) using Atlantic salmon smolts (initial weight 242.1 ± 46.0 g; initial length, 27 ± 1.8 cm) from the Saint John River stock. The fish were received from Cooke Aquaculture (St. Alban’s, NL, Canada), where they were held in freshwater, and were transferred to seawater at the JBARB to undergo smoltification. Six hundred fish were randomly distributed into twelve 500-L tanks (50 fish per tank) with flow-through water supply at a rate of 12 L min^−1^ (temperature, 14 °C; dissolved oxygen, 10 mg L^−1^) and all fish were kept on a photoperiod of 12-h light and 12-h dark. Fish were fed a commercial diet (Nutra Transfer NP, 3 mm, Skretting Canada, St. Andrews, NB, Canada) during a 1-week acclimation period. Once the fish were acclimated, they were gradually moved onto the control diet over a period of 3 days. Initial sampling was done 1 week after the fish were completely weaned from the commercial diet. Subsequently, fish were weaned onto their assigned experimental diet (control, 8CM, 16CM, or 24CM) over another period of 3 days. Fish were fed the experimental diets to apparent satiety twice daily in triplicate tanks over a period of 16 weeks.

### Tissue Sampling

At each sampling time point fish were removed from their tank using a net and over-anesthetized in 400 mg L^−1^ tricaine-methane-sulfonate bath (TMS; Syndel Laboratories, Vancouver, BC, Canada). Three fish per tank were dissected by removing the total viscera, and the DI was removed. For each fish, an ~2-cm-long section was removed from the middle of the DI; the distal-most ~0.5-cm portion was flash-frozen for RNA isolation (i.e., placed in RNase-free 1.5-mL microcentrifuge tubes, flash-frozen in liquid nitrogen and placed on dry ice until they could be moved to a −80 °C freezer for long-term storage), and the remainder was fixed for histological analyses (i.e., stored in 10 % formalin at room temperature). Further details on the rearing conditions and sampling can be found in Hixson et al. (2014, 2016), which focused on lipid analyses of diets and tissues and did not include gene expression analyses.

### RNA Extraction, DNAse-I Treatment, and Column Purification

Frozen tissue samples were homogenized using ceramic mortars and pestles, which were previously cleaned with bleach and baked at 220 °C for 5 h to inactivate RNases. Homogenization was done under liquid nitrogen; after the addition of TRIzol reagent (Invitrogen, Carlsbad, CA, USA), samples were further homogenized using QIAshredders (QIAGEN, Mississauga, ON, Canada) and RNA was extracted following the manufacturers’ instructions. Twenty micrograms of total RNA were treated with 6.8 Kunitz units DNaseI (RNase-free DNase kit, QIAGEN) to remove genomic DNA. DNaseI-treated RNA samples were column-purified using the RNeasy MinElute Cleanup Kit (QIAGEN) following the manufacturer’s protocol. RNA integrity and purity were assessed using 1 % agarose gel electrophoresis, and A260/230 and A260/280 NanoDrop spectrophotometry, respectively.

### Microarray Hybridization and Scanning

**Fig. 1:**
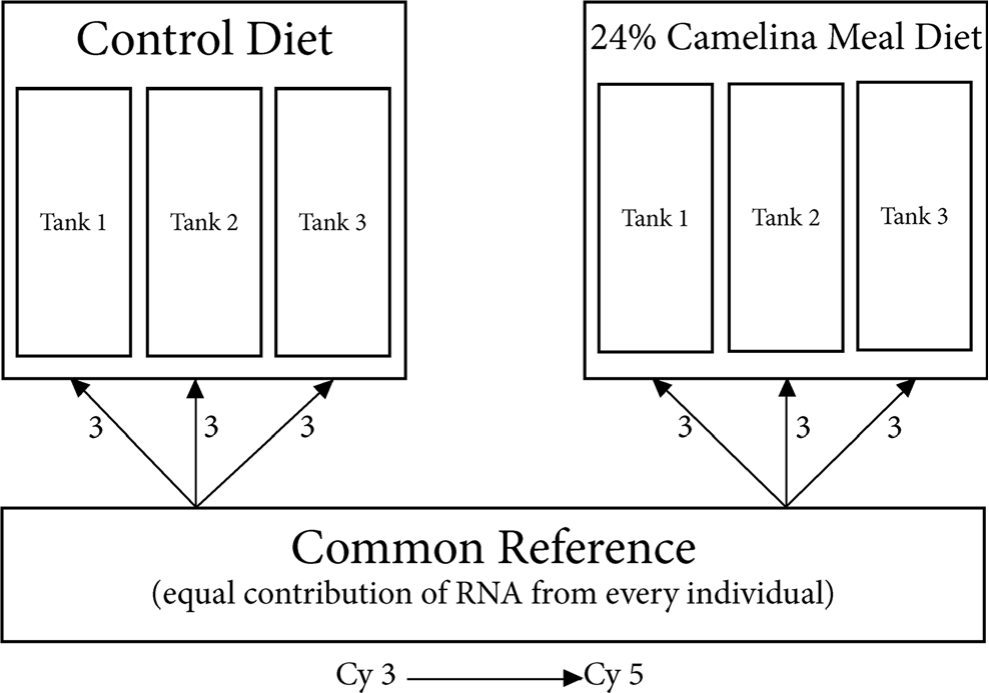
Microarray experimental design. *Arrows* indicate arrays with number of biological replicates indicated by *numbers next to the arrows*. Eighteen arrays were used in total

The experimental microarray design can be seen in Fig. 1. Three individuals from each of the three replicate tanks (*n* = 9) from the control diet (no CM inclusion) and from the 24CM (high level CM inclusion diet) were used for microarray analysis with the 4x44K Atlantic salmon oligonucleotide array (Agilent Technologies, Mississauga, ON, Canada; Jantzen et al. 2011).

Microarray hybridization was carried out following the protocols outlined in Xue et al. (2015). Microarrays were hybridized using labeled antisense amplified RNA (aRNA) synthesized from 1 μg of DNaseI-treated, column-purified RNA that had satisfactory purity measurements (i.e., A260/230 and A260/280>1.8) and showed tight 18S and 28S ribosomal RNA bands (i.e., high integrity). A “common reference” sample was generated by pooling 5 μg of RNA from each of the 18 samples so that it included RNA from all of the samples analyzed. The concentration, purity, and integrity of the pooled RNA sample were checked as previously described. For each of the experimental samples or the common reference pool, 1 μg of DNaseI-treated, column-purified RNA was used to synthesize aRNA using the Amino Allyl MessageAmp II aRNA Amplification Kit (Life Technologies, Burlington, ON, Canada) following the manufacturer’s instructions. Briefly, 1 μg of DNaseI-treated, column-purified RNA was reverse transcribed to cDNA which was used as a template for an in vitro transcription reaction using amino allyl modified dUTP. Twenty micrograms of aRNA were precipitated overnight, re-suspended in coupling buffer, and labeled with either Cy5 dye (experimental samples) or Cy3 dye (common reference) (GE Healthcare, Baied’Urfe, QC, Canada) following the manufacturer’s instructions. Labeled aRNA from one experimental sample was pooled together with the common reference and, for each target, 825 ng of labeled aRNA was fragmented using 25X fragmentation buffer as well as 10X blocking agent (Agilent Technologies) and RNAse-free dH_2_O. These were co-hybridized to a consortium for Genomic Research on All Salmonids Project (cGRASP)-designed Agilent 4x44K salmonid oligonucleotide array (GEO accession: GPL11299) (Jantzen et al. 2011; Sahlmann et al. 2013) as per the manufacturer’s instructions. Hybridizations were carried out at 65 °C for approximately 16 h with 10 rpm rotation in an Agilent hybridization oven (Jantzen et al. 2011). The arrays were washed immediately in Agilent Gene Expression Wash buffer I on a rocker platform for 5 min at room temperature, then washed in Agilent Gene Expression Wash Buffer II on a rocker platform for 5 min at 37 °C. The arrays were dried by centrifuging in 50-mL tubes at 200 rpm for 5 min at room temperature.

The arrays were immediately scanned using a ScanArrayGx Plus scanner (Perkin Elmer, Waltham, MA, USA) at a resolution of 5 μm. Photomultiplier tube (PMT) settings were adjusted for each array individually with the laser power set at 90 % for both channels (Cy3 and Cy5). Scanned array images were saved in TIFF format to be used for data extraction.

Scanned images were processed in the Imagene v9.0 software (BioDiscovery, El Segundo, CA, USA) using the cGRASP 4x44K GAL file to extract probe intensities. The extracted data were imported into the R statistical computing software where background correction, data transformation (Log_2_), print-tip Loess normalization and removal of control and flagged (low-quality) spots were carried out. This was performed using the Bioconductor and mArray packages for R, using scripts adapted from those reported in Booman et al. (2011). After spot quality filtering, spots absent on more than 30 % of the arrays (i.e., 5 out of 18 arrays) were rejected, which resulted in a final list of 14,897 probes for analysis. The microarray data has been submitted to the Gene Expression Omnibus (GEO) under the accession number GSE68732.

### Microarray Data Analyses

Prior to significance analysis of microarrays (SAM) and rank products (RP) analyses, missing data points for the 14,897 probes were imputed using the EM_array method from LSimpute (Bo et al. 2004; Chiu et al. 2013). A reference design comparison was carried out between the control group and the 24CM group using the SAM algorithm (Tusher et al. 2001) to identify any genes that were significantly differentially expressed between the groups. SAM analysis was performed at a false discovery rate (FDR) threshold of 6.25 % using Bioconductor in the siggenes package for R. The resulting gene list was re-annotated using the expressed sequence tag (EST) information or contiguous sequence (contig) from which the 60mer oligonucleotide array probes were designed. Annotation was done manually with a BLASTX alignment against the NCBI non-redundant (nr) database using an *E* value threshold of 10^−5^. To functionally annotate the microarray-identified transcripts, gene ontology (GO) terms were manually obtained using the putative ortholog from *Homo sapiens* (i.e., best human BLASTX hit) from the UniProt Knowledgebase (http://www.uniprot.org).

In order to confirm the results obtained using SAM and to potentially identify additional transcripts that were differentially expressed between 24CM and control groups, the microarray data were also analyzed using the RP method (Breitling et al. 2004). RP is a statistical approach whose ability to identify differentially expressed genes is less affected by high variability and low biological replication (Breitling et al. 2004; Jeffery et al. 2006). RP analysis was performed at a percentage of false-positives (PFP) threshold of 5 % using the Bioconductor package, RankProd (Hong et al. 2006). The resulting gene lists were annotated in Blast2GO (Conesa et al. 2005) using the BLASTX algorithm against the nr protein database of NCBI (2015.09.30). The best BLASTX hit that had an *E* value < 10^−5^ and an informatively named protein product was chosen. Gene Ontology (GO) terms mapped to each microarray probe in Blast2GO were collected.

### Real-Time Quantitative Polymerase Chain Reaction (qPCR)

Complimentary DNA (cDNA) was synthesized from 1 μg of column-purified total RNA in 20 μl reactions using 250 ng of random hexamers (Life Technologies), 1 μL of dNTPs (10 mM each), and Moloney-Murine Leukemia Virus (M-MLV) reverse transcriptase (Life Technologies) with the first strand buffer and Dithiothreitol (DTT) supplied by the manufacturer. Samples were incubated as follows: 65 °C for 5 min after the addition of the random hexamers and dNTPs, then 25 °C for 10 min and 37 °C for 50 min after the addition of the first-strand buffer, DTT, and M-MLV. cDNA was diluted 1:10 using nuclease-free water (Gibco, Life Technologies). Negative controls were performed by omitting the reverse transcriptase.

Candidate genes from the microarray analysis were subjected to qPCR to validate transcript levels. cDNA was synthesized from nine individuals (three from each replicate tank) from each of the four experimental diets (control, 8CM, 16CM and 24CM) for a total of 36 individuals. Primers used for qPCR analysis can be found in Table 2. All primers were quality checked (QC) before use. qPCR primer QC included running five-point 1:3 dilutions using pooled cDNA from all samples representing a starting quantity of 10 ng of input total RNA. This was done to determine the amplification efficiency of each primer pair (*E* = 10^[−1/slope]^; Pfaffl 2001). Melt curves were also performed (+1 % increases over 30 s from 60 to 95 °C) to verify that only a single product was being amplified and that there was no dimerization of primers. This was also done to verify that there was no amplification in the no-template controls (i.e., to confirm the absence of contamination).

**Table 2:** Primers used for qPCR analysis of microarray-identified transcripts responsive to camelina meal-containing diets in Atlantic salmon

Gene name (symbol)		Primer sequence (5′–3′)	Sequence used for primer design	Amplification efficiency (%)	Amplicon size (bp)
*ES1 protein homolog* (*es1*)	F	ACTATAAAGGGAACAGGGTGCC	EB085110	89.3	167
	R	CAAGCTGGTCACCTCCTCTG			
*transmembrane protease serine 9-like* (*tmprss9*; alias: polyserase-I)	F	TAGTGCCGTTAGTGCTGGTG	EG876684	95.8	194
	R	CCAGACCTCTGTGGTGGAAT			
*thioredoxin* (*txn*)	F	GGATTCCTTCTTCAGTGCCC	BT049355	90.1	196
	R	GATGTCACAGTGTTTGGCCA			
*pirin* (*pir*; alias: probable quercetin 2,3-dioxygenase)	F	GAGGTCTAAGATCAGGGATTCC	NM_001141576	102.6	200
	R	GGAGGCACAGTGACAAAACA			
*ependymin* (*epd*)	F	TGACTGGAGCCATGTCAGTG	BT058166	99.4	125
	R	CAGGCCGAATGTCTTGTTCT			
*N-acyl-phosphatidylethanolamine-hydrolyzing phospholipase D* (*napepld*)	F	AAGACATCCAGGCCAGACAC	DY730770	99.9	126
	R	GCTCCACATTCAACCCATTC			
*gamma-interferon-inducible lysosomal thiol reductase* (*GILT)*	F	AAATGGGGAGCACACAGAAG	BT047373	100.2	105
	R	GCCGTACTACAGGCTTCAGG			
*matrix metalloproteinase 13* (*mmp13*)	F	ACGATGACGAGTCCTTCAGC	DW539943	102.2	153
	R	AGGTGCTGGGGTTTGTGTAG			
*transforming growth factor β* (*tgfb*)	F	AGTTGCCTTGTGATTGTGGGA	EU082211	108.0	191
	R	CTCTTCAGTAGTGGTTTGTCG			
*elongation factor 1 alpha-2* (*ef1a2*; normalizer)	F	TCGAGTGAGCGCACAGTAAC	GO064943	103.6	123
	R	CCATCTCAGCTGCTTCCTTC			
*60S ribosomal subunit L32* (*rpl32*; normalizer)	F	AGGCGGTTTAAGGGTCAGAT	BT043656	102.4	119
	R	TCGAGCTCCTTGATGTTGTG			

Primers were quality checked using the reference cDNA as the template (see Materials and Methods for details)

qPCR analysis was carried out using the ViiA7 real-time PCR system and SYBR Green I dye chemistry (Applied Biosystems, Foster City, CA, USA) with the following conditions: 50.0 °C for 5 min, pre-incubation at 95.0 °C for 10 min, and 40 cycles of 95 °C for 15 s and 60 °C for 1 min. Each amplification was performed using 13 μl reactions with 4 μl of input cDNA (representing 10 ng of input RNA), 1.46 μl of PCR-grade water (Life Technologies), 6.5 μl of 2x SYBR Green Mastermix (Applied Biosystems) and 0.52 μl each of the forward and reverse primers (1.25 μM concentration). Expression levels were normalized to *elongation factor 1 alpha* (*ef1a*) and *60S ribosomal rna subunit L32*. Five candidate normalizer genes were tested across 18 samples using the GeNorm software (Vandesompele et al. 2002) (BioGazelle, Zwijnaarde, Belgium) and *ef1a* and *60S ribosomal rna subunit L32* were selected as normalizer genes based on their high expression stability across all samples (GeNorm V < 0.15, average GeNorm M < 0.05). Expression analyses were carried out in 384-well format with each sample run in triplicate. The respective C_T_ values were determined using the ViiA7 Gene Expression Study Application (Applied Biosystems) with automated threshold determination and walking baseline. The relative starting quantity (RQ) was determined for each transcript using the 2^−ΔΔCT^ method (Livak and Schmittgen 2001) using the sample with the lowest expression for each gene of interest as a calibrator. Amplification efficiencies (Table 2) were used to calculate each RQ value.

### Histology

DI tissue was prepared from fish from the week-16 time point using standard histological techniques. Briefly, samples were dehydrated in ethanol, cleared using xylene and infiltrated with paraffin. Samples were embedded in paraffin blocks and 4 μm cross-sections were taken (i.e., across the lumen of the intestine). All slides were stained using hematoxylin and eosin, and blinded microscopic evaluations were carried out (i.e., fish were evaluated without knowing from which experimental group they came).

Quantitative evaluations were performed to determine physiological changes in mucosal fold height, LP width, presence of GCs, infiltration of EGCs in the LP and SEM thickness. For mucosal fold height, measurements were taken from the tip of the mucosal fold to the bottom of the epithelial cells at the base of the fold. The LP width was measured at the top of the fold at the end of the LP, at the midpoint of the mucosal fold, and at the base of the mucosal fold, and an average was taken for each villus. GCs and EGCs were counted and normalized to the area of the mucosal fold and LP, respectively. Three fish were assessed from each tank with 10 simple villi from a single section being examined from each fish. SEM width was measured at three points per fish: 0°, 120°, and 240° around the lumen (three fish per tank, *n* = 9). All quantitative measurements were made using the ImageJ image analysis software (Schneider et al. 2012). A semi-quantitative scoring system, described in Uran et al. (2008), was used to measure the degree of supranuclear vacuolization. Briefly, a scoring system ranging from 1 to 5 was used, with 1 being a normal intestinal state (i.e., high level of vacuolization in the enterocytes) and 5 being the most extreme inflammation state (i.e., complete extinction of vacuolization in the enterocytes).

### Statistical Analyses

Statistical analysis of growth and histology data was carried out using Minitab 16 (Minitab Inc., State College, PA, USA). A two-way nested ANOVA was performed using the general linear model with a term that accounts for the effect of diet on the response variable and a term that nests tanks within diets to negate any tank effect or individual variance that may be present. Tukey’s post hoc tests for multiple comparisons at a significance of 5 % (i.e., *p* < 0.05) were carried out for pairwise comparisons.

RQ values obtained from the Applied Biosystems Gene Expression Study Application were statistically analyzed using GraphPad Prism version 6.0 (La Jolla, CA, USA). Data were tested for normality using the Shapiro–Wilks test and analyzed using a one-way ANOVA and Tukey’s multi-comparison pairwise post hoc test. Statistically significant difference in RQ values between test groups was taken at 5 %.

Correlation between histopathological parameters (except villus height), or between histopathological parameters and transcript expression data, was evaluated using Pearson’s correlation coefficients using MiniTab 16.

## Results

### Growth Performance

The growth performance of the fish involved in this feeding trial was previously reported in Hixson et al. (2016), and the growth performance of fish fed the control diet (0CM) was also reported in Hixson et al. (2014); however, the results for the subset of diets that were used in this study are briefly described and reported herein as they are directly pertinent to the current study. The Atlantic salmon grew from an initial weight of 230–246 g to a final weight of 565–591 g. The fish groups fed any of the CM-containing diets in this study gained less weight and had a lower final weight than the fish group fed the control diet after the 16-week feeding trial was completed (*p* = 0.003 and *p* = 0.001, respectively). The apparent feed intake was decreased by the CM-containing diets compared to the FM control (*p* = 0.001). However, fish fed all diets had comparable feed conversion ratios (*p* = 0.299). The visceral somatic index (VSI) of fish fed the CM-containing diets also increased when compared to the fish fed the control diet (*p* = 0.001) but there was no significant difference between the CM diet groups. The growth performance of the fish, as measured by the specific growth rate, remained unchanged by the CM-containing diets (*p* = 0.107). Full growth performance results are reported in Table 3 and additional details about growth performance for the fish in this trial, as well as the other groups involved in this feeding trial (i.e., those that were not analyzed in this study) are reported in Hixson et al. (2014, 2016).

**Table 3:** Week 16 growth performance of Atlantic salmon fed a control diet or camelina meal (CM)-containing diets

Growth parameter	0CM	8CM	16CM	24CM	*p* value
Initial weight (g)^a^	230 ± 41	246 ± 62	250 ± 42	241 ± 49	0.637
Final weight (g)^a^	691 ± 153a	576 ± 152b	560 ± 129b	565 ± 117b	0.001
Weight gain (g)^b^	471 ± 39a	329 ± 72b	309 ± 45b	327 ± 17b	0.003
Initial length (cm)^a^	26.2 ± 2.4	26.9 ± 1.9	27.5 ± 1.5	26.8 ± 1.6	0.424
Final length (cm)^a^	35.0 ± 4.1	33.8 ± 2.8	33.4 ± 2.6	33.3 ± 2.9	0.081
SGR (%/day)^b^	0.99 ± 0.1	0.77 ± 0.2	0.72 ± 0.1	0.76 ± 0.01	0.107
CF^a^	1.53 ± 0.1	1.46 ± 0.1	1.48 ± 0.1	1.54 ± 0.5	0.106
VSI (%)^c^	9.8 ± 1.1a	10.8 ± 1.0b	11.2 ± 1.2b	11.4 ± 0.9b	0.001
AFI^d^	515 ± 7.6a	420 ± 57b	384 ± 33b	391 ± 20b	0.001
FCR^d^	1.01 ± 0.1	1.20 ± 0.2	1.16 ± 0.1	1.10 ± 0.1	0.299

The growth results from this trial were previously reported in Hixson et al. (2016)

All measurements are presented as mean ± SD. Means with different letters are significantly different (*p* < 0.05)

^a^Weight, length, and condition factor (*CF*) measurements were calculated from individual fish. Initial measurements: *n* = 9; final measurements: *n* = 48 (control), 66 (8CM), 69 (16CM), 50 (24CM). *CF* was calculated as described in Hixson et al. (2016)

^b^Specific growth rate (SGR) = (ln(Final weight) − ln(Initial weight)/No. of days in trial) × 100. Weight SGR and weight gain (g fish^-1^) calculated using tank means, *n* = 3

^c^Visceral somatic index (%) = 100 × (Viscera mass/Body mass) *n* = 27

^d^Apparent Feed Intake (AFI) (g fish^-1^) and Feed Conversion Ratio (FCR) were calculated as described in Hixson et al. (2016) using tank means, *n* = 3

### Intestine Transcriptome Analysis

A microarray experiment utilizing nine biological replicates was carried out to identify transcripts responsive to a CM-containing diet (24CM) compared to a control diet in the DI of Atlantic salmon. SAM analysis (FDR 6.25 %) identified 16 differentially expressed features, with all being more highly expressed in the fish fed the 24CM diet; the fold change values of these microarray-identified genes ranged from 1.57 to 3.94. In total, eight different transcripts were represented by the SAM-identified features and six were annotated through BLASTx searches against the NCBI nr protein database (Table 4). RP detected a total of 136 differentially expressed features with a PFP threshold of 5 % (82 more highly expressed in the salmon fed 24CM diet and 54 more highly expressed in the control diet) (Electronic Supplementary Material (ESM), Supplemental Tables 1 and 2). Twelve of the 16 SAM-identified features were also represented in the RP lists (Table 4). The remainder of the Results, as well as the Discussion, will focus mainly on the genes that were identified as CM responsive using both SAM and RP (with reported fold change values calculated using SAM).

**Table 4:** Microarray-identified transcripts that were significantly upregulated in salmon fed the 24CM diet compared to salmon fed the control diet

Probe ID^a^	Fold change^b^	BLASTx identification^c^				Gene ontology (GO) of putative human ortholog^d^
		Best named BLASTx hit [species]	Accession no.	*E* value	% ID (AA)	
**C011R096**	3.33	ES1 protein like protein (Es1) [*Chelonia mydas*]	EMP32012	3e-12	27/39 (69 %)	Mitochondrion (CC)
**C215R002**	2.59	PREDICTED transmembrane protease serine 9-like (Tmprss9) [*Stegastes partitus*]	XP_008289151	3e-29	71/133 (53 %)	Proteolysis (BP), catalytic activity (MF) serine-type endopeptidase activity (MF), hydrolase activity (MF), integral component of plasma membrane (CC)
**C227R083**	2.46	Thioredoxin (Txn) [*Salmo salar*]	ACM09155	2e-68	107/108 (99 %)	Negative regulation of transcription from RNA polymerase II promoter (BP), Response to reactive oxygen species (BP), Glycerol ether metabolic process (BP), Movement of cell or subcellular component (BP), Signal transduction (BP), Cell proliferation (BP), Response to radiation (BP), Nucleobase-containing small molecule interconversion (BP), Activation of protein kinase B activity (BP), Positive regulation of peptidyl-serine phosphorylation (BP), Translocation (BP), Nucleotide-binding domain (BP), Leucine rich repeat containing receptor signaling pathway (BP), Positive regulation of DNA binding (BP), Innate immune response (BP), Cell redox homeostasis (BP), Negative regulation of hydrogen peroxide-induced cell death (BP), Protein binding (MF), Protein disulfide oxidoreductase activity (MF), Poly(A) RNA binding (MF), Extracellular region (CC), Nucleus (CC), Cytoplasm (CC), Mitochondrion (CC), Extracellular vesicular exosome (CC).
**C075R139**	2.25					
**C203R158**	2.27					
**C040R065**	2.04					
*C182R133*	1.97					
*C050R109*	1.89					
**C110R097**	1.92					
**C251R109**	3.94	Pirin (Pir) [*Salmo salar*]	NP_001135048	7e-69	107/115 (93 %)	Transcription, DNA-templated (BP), myeloid cell differentiation (BP), monocyte differentiation (BP), oxidation–reduction process (BP), transcription cofactor activity (MF), protein binding (MF), quercetin 2,3-dioxygenase activity (MF), oxidoreductase activity (MF), metal ion binding (MF), nucleus (CC), cytoplasm (CC)
*C230R082*	1.89	Ependymin (Epd) precursor [*Salmo salar*]	ACM09231	1e-62	111/112 (99 %)	Cell-matrix adhesion (BP), calcium ion binding (MF), extracellular region (CC), lysosome (CC), extracellular vesicular exosome (CC)
**C210R115**	2.00					
*C005R058*	1.57					
**C231R006**	2.81	PREDICTED: N-acyl-phosphatidyl ethanolamine-hydrolyzing phospholipase D-like (Napepld) [*Lepisosteus oculatus*]	XP_006633527	2e-75	108/143 (76 %)	Lipid metabolic process (BP), phospholipase activity (MF), zinc ion binding (MF), hydrolase activity (MF), N-acylphosphatidylethanolamine-specific phospholipase D activity (MF), membrane (CC), photoreceptor outer segment membrane (CC), extracellular vesicular exosome (CC)
**C034R078**	2.02	Unknown	N/A	N/A	N/A	N/A
**C202R142**	3.88	Unknown	N/A	N/A	N/A	N/A

The 12 microarray features that were identified as significantly upregulated in 24CM using both SAM and RP (see Materials and Methods for details) are shown with probe IDs in bold font. The remaining four features were identified by SAM (but not RP) as upregulated in 24CM, and are shown with probe IDs in non-bold italics. All 82 features identified by RP as upregulated in 24CM are available in ESM Supplemental Table 1, and all 54 features identified by RP as downregulated in 24CM are available in ESM Supplemental Table 2

^a^Represents the identity of the associated probe on the 4x44K Atlantic salmon microarray. Some genes were represented by several probes on the microarray

^b^Fold change values of individual probes are reported

^c^Genes were identified by BLASTX using the contig from which the microarray probe was designed as the query sequence against the non-redundant protein database of NCBI. The best BLASTX hit that had an expect (*E*) value < 10^−5^ and an informatively named protein product was chosen and is represented in this table along with the associated accession number and the species identification

^d^Gene Ontology (GO) terms associated with the putative ortholog from *Homo sapiens* (i.e., the best human hit) are shown. Redundant GO terms were replaced with a single representative term. GO terms are separated into three categories: Biological Process (BP), Molecular Function (MF) or Cellular Component (CC). Uniprot accession numbers for putative human orthologs are as follows: ES1 protein like protein: P30042, transmembrane protease serine 9-like: Q7Z410, Thioredoxin: P10599, Pirin: O00625, Ependymin: Q9UM22, N-acyl-phosphatidylethanolamine-hydrolyzing phospholipase D-like: Q6IQ20

The differentially expressed genes identified by the microarray included *ES1 protein homolog* (*es1*), which was identified by one common (i.e., identified by both SAM and RP analyses) feature that was 3.3-fold upregulated (Table 4). The only GO term associated with this gene was “mitochondrion”. *Transmembrane protease serine 9* (*tmprss9*) was identified by one common differentially expressed feature (2.6-fold upregulated) and GO annotation included “proteolysis” and “integral component of plasma membrane”. *Thioredoxin* (*txn*) was represented by five common upregulated features (1.9- to 2.5-fold) and had many functional annotations, including “cell proliferation”, “innate immune responses”, “cell redox homeostasis”, and “negative regulation of hydrogen peroxide induced cell death”. *Pirin (pir*) was represented by one common upregulated feature (3.9-fold) and was associated with GO terms including “myeloid cell differentiation”, “monocyte differentiation” and “oxidoreductase activity”. *Ependymin* (*epd*) was represented by one common upregulated feature (2.0-fold). The functional annotation for this gene included the GO terms “cell matrix adhesion” and “calcium ion binding”. The last gene identified by both SAM and RP with a significant BLASTX hit was *N-acyl-phosphatidylethanolamine-hydrolyzing phospholipase D* (*napepld*), which was represented by one common upregulated feature (2.8-fold) and included the GO terms “phospholipase activity” and “zinc ion binding”. Two features were identified by both SAM and RP that did not have significant BLASTX hits, meaning that their identities could not be determined for this study. These genes were represented by one common feature each and had fold changes of 2.0 and 3.9 (Table 4).

### qPCR Validation

**Fig. 2:**
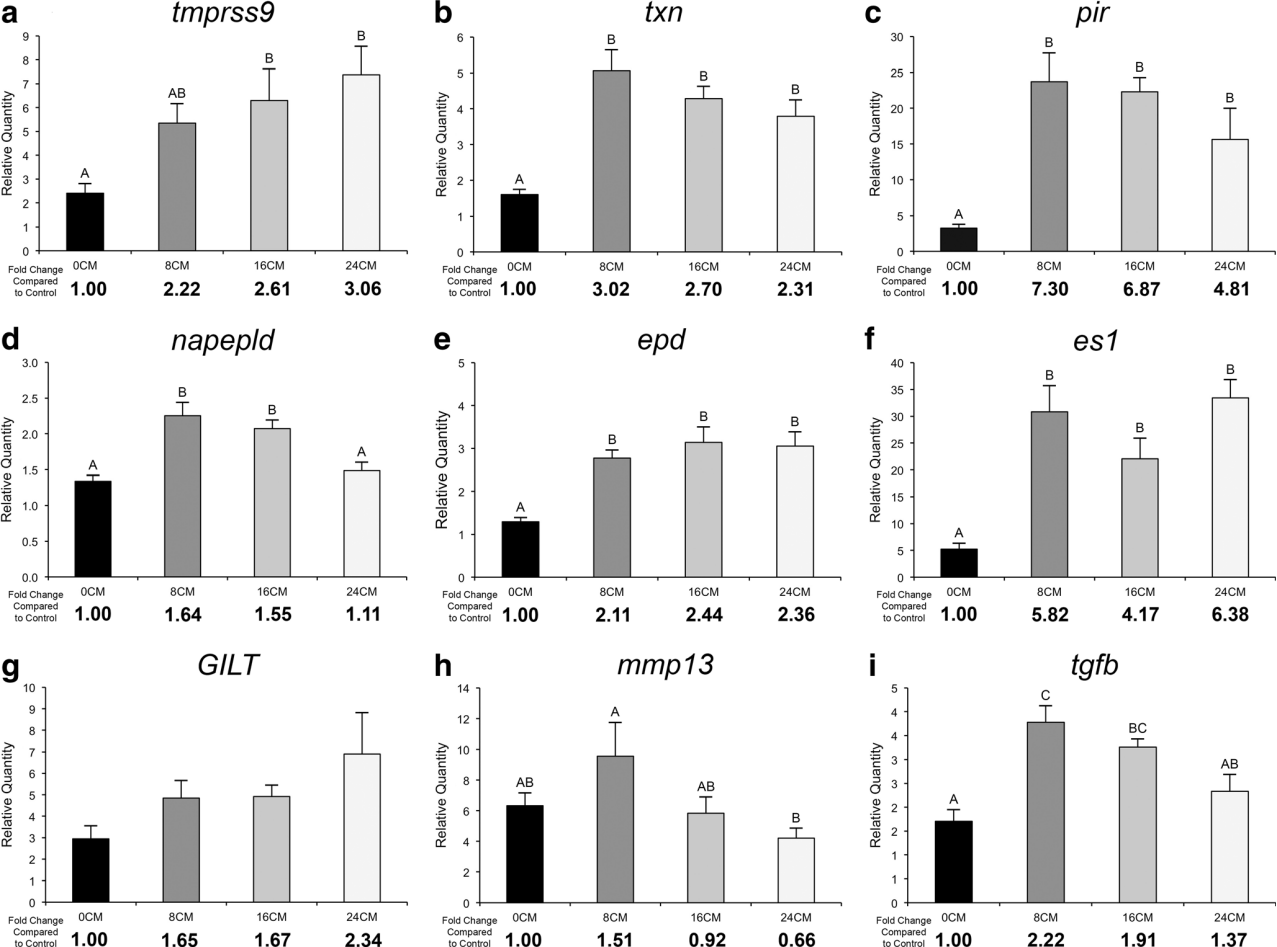
qPCR validation of microarray-identified transcripts. Relative transcript levels were measured in the hindguts of fish fed the control diet and the camelina meal-containing test diets (8CM, 16CM, and 24CM) at week 16. Transcript relative quantity (RQ) values taken as mean RQ data + SE. *Bars with different letters* are significantly different (*p* < 0.05). Upregulation was calculated as *A*/*B* where *A* is the mean RQ from an experimental group and *B* is the mean RQ from the control group

The expression of the six transcripts identified by both SAM and RP, and with significant BLASTX hits, was quantified using qPCR (Fig. 2a–f). This was done to validate the upregulation of the genes in the 24CM group and also to measure the expression of these genes in the fish fed the 8CM and 16CM diets. Five of the six genes (i.e., all except *napepld)* were validated to be responsive to the 24CM diet. All six genes were significantly upregulated in the fish fed the 16CM diet compared to the fish fed the control diet, and five out of the six genes (i.e., all except *tmprss9*) were upregulated in the fish fed the 8CM diet compared to those fed the control diet.

Additional qPCR assays were designed (see Table 2 for primers) for three inflammatory biomarker genes that are typically responsive during acute food-mediated enteritis in Atlantic salmon (Marjara et al. 2012; Sahlmann et al. 2013) and were not identified by the microarray in the current study. These genes include *gamma-interferon-inducible lysosomal thiol reductase* (*GILT*), *matrix metalloproteinase 13* (*mmp13*), and *transforming growth factor beta* (*tgfb*) (Fig. 2g–i). Of these additional genes, *tgfb* was significantly upregulated by the 8CM and 16CM diets compared with fish fed the control diet, and *mmp13* was significantly downregulated by the 24CM diet (*p* = 0.050) compared with fish fed the 8CM diet (Fig. 2h, i).

### Histological Analysis

**Fig. 3:**
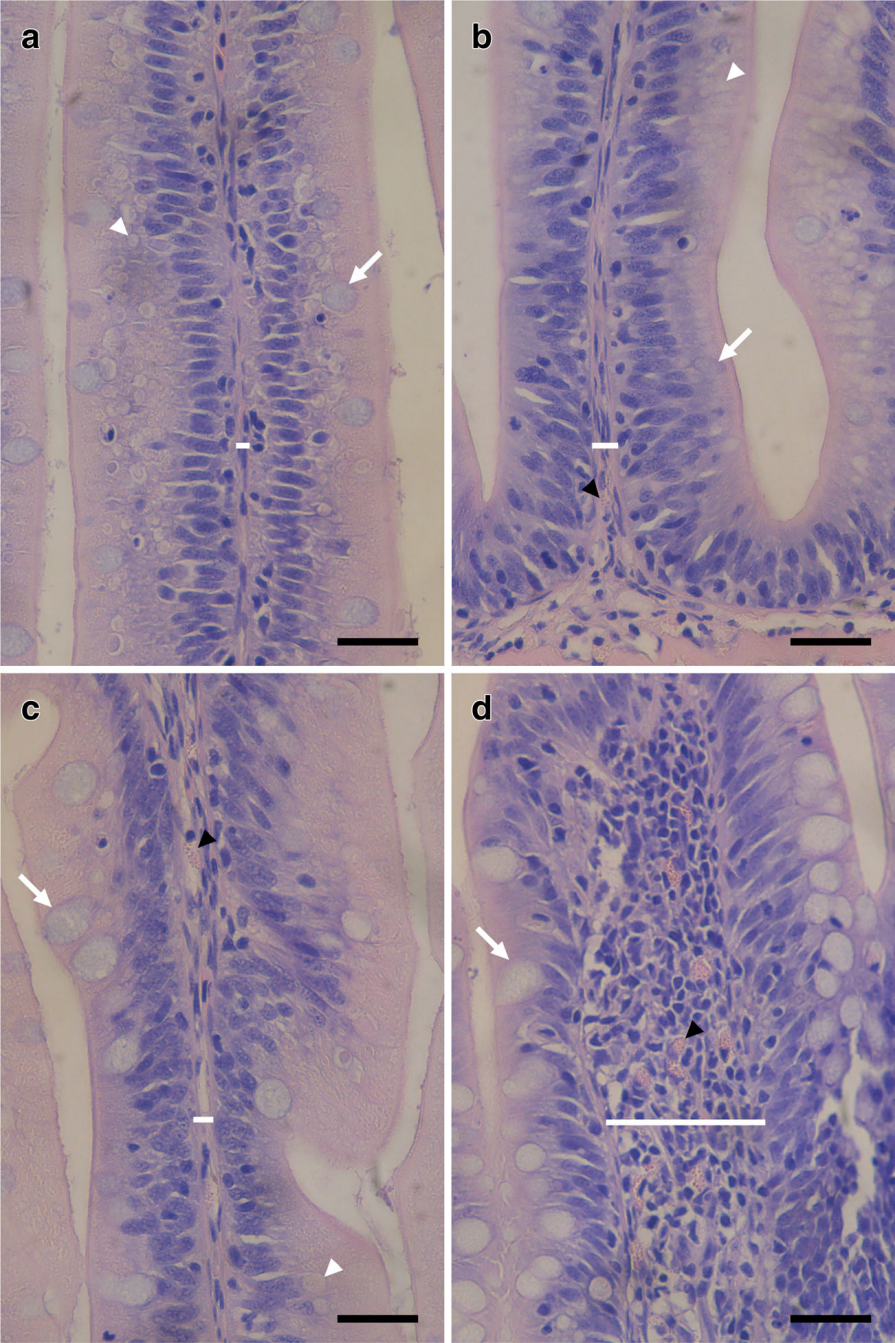
Light micrographs of villi from the DI of Atlantic salmon showing histopathological changes in the DI of Atlantic salmon fed either a control FM-containing diet or one of an 8CM, 16CM, or 24CM diet. **a** 0CM, **b** 8CM, **c** 16CM, **d** 24CM. *White lines* indicate lamina propria width, *white arrows* indicate goblet cells, *white arrow heads* indicate absorptive vacuoles, *black arrow head* indicate eosinophilic granule calls, *black size bars* represent 25 μm

**Fig. 4:**
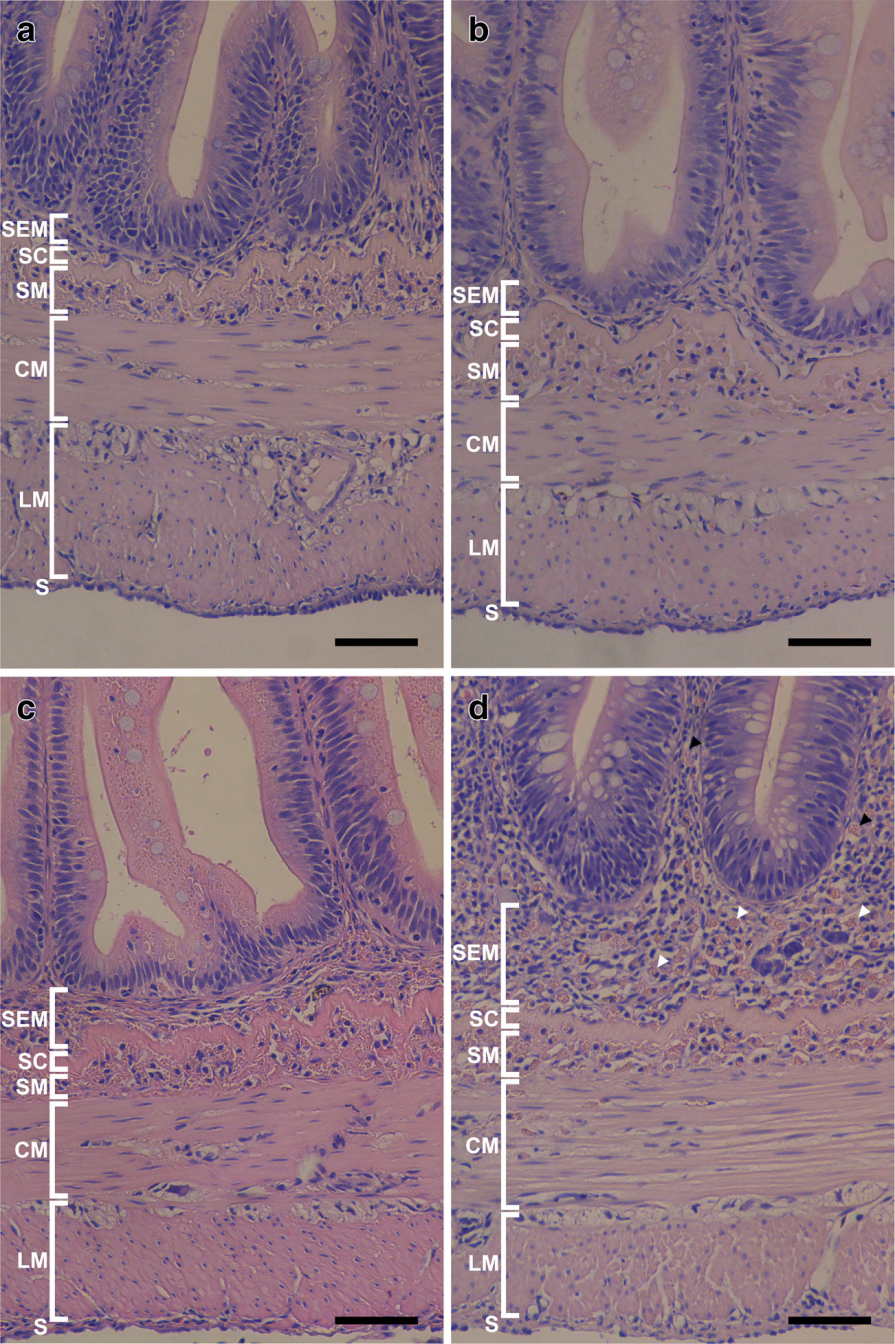
Light micrographs of DI walls of Atlantic salmon fed either a control FM-containing diet or one of an 8CM, 16CM, or 24CM diet. **a** 0CM, **b** 8CM, **c** 16CM, **d** 24CM. *M* = mucosa, *SEM* = sub-epithelial mucosa, *SC* = stratum compactum, *SM* = sub mucosa, *CM* = circular muscles, *LM* = longitudinal muscles, *S* = serosa. *Black size bars* represent 100 μm. Thickening of SEM, as well as increased infiltration of EGCs into the SEM and the LP can be seen in **d** (24CM). *Black arrowheads* indicate EGCs that have infiltrated in to the LP, *white arrowheads* indicate resident EGCs in the SEM

Morphological changes in the Atlantic salmon DI were observed at the week 16 time point as a result of the dietary inclusion of CM and are reported in Table 5. Quantitative analyses indicate no significant change in the height of the villi; however, a significant widening of the LP was seen in the fish fed the 24CM diet compared to all other diets with no significant change in the LP width in the fish fed the 8CM or 16CM diets compared to control (*p* = 0.001). There was a significant increase in the number of GCs per square millimeter in the DI of the fish fed the 24CM diet compared to all other diets and in fish fed 16CM diets compared to the 8CM diet and control (*p* = 0.027). There was no significant change in the number of GCs per square millimeter seen in the fish fed the 8CM diet compared to the control diet. The counts of EGCs that had infiltrated into the LP showed a significant increase seen in the fish fed the 24CM diet compared to all other diet groups (*p* = 0.004); however, there was no significant change in the number of EGCs that had infiltrated into the LP seen in the fish fed the 8CM or 16CM diet compared to the control group. Images showing increased EGCs and GCs can be seen in Fig. 3. It was also noted that the population of EGCs in the SEM appeared to increase in density with an increase in dietary CM inclusion, as seen in Fig. 4. In the fish fed the 16CM diet, there was a significantly decreased amount of SNV when compared to fish fed the control and 8CM diets and a significant decrease seen in SNV in fish fed the 24CM diet when compared to all other diets (*p* = 0.001). This is illustrated with representative images in Fig. 3. The SEM was significantly thickened in fish fed the 24CM diet when compared to all other diets (*p* = 0.046), although there was no notable change in the fish fed the other CM-containing diets compared to the control (Table 5, Fig. 4). It was also noted that many fused villi were seen in the fish fed the 24CM diet; although not quantified, villus fusion appeared to be much reduced in the fish fed the other experimental diets. All of these morphological changes are consistent with the presence of intestinal inflammation in Atlantic salmon.

**Table 5:** Quantitative^a^ histopathological analysis of the DI of Atlantic salmon fed CM-containing diets for 16 weeks

	0CM	8CM	16CM	24CM	*F* stat	*p* value
Villus height (μm)	482.73 ± 193.12	544.79 ± 281.67	397.53 ± 174.77	454.51 ± 215.61	2.71	0.115
Lamina propria width (μm)	13.94 ± 8.05bc	17.14 ± 14.83b	11.89 ± 6.86c	24.56 ± 23.36a	29.00	0.001
SEM width (μm)	11.94 ± 4.45b	16.23 ± 6.08b	20.66 ± 10.34b	39.95 ± 25.10a	4.20	0.046
Eosinophilic granular cells (cells/mm^2^)	42.26 ± 118.41b	75.52 ± 156.25b	204.73 ± 361.91b	550.89 ± 594.81a	9.95	0.004
Goblet cells (cells/mm^2^)	347.95 ± 235.83c	306.41 ± 128.69c	495.70 ± 311.76b	793.23 ± 409.65a	5.24	0.027
Supranuclear vacuolization^a^	1.22 ± 0.42c	1.28 ± 0.45c	1.72 ± 0.60b	2.91 ± 0.65a	113.96	0.001

All measurements are presented as mean ± SD. Means with different letters are significantly different letters (*p* < 0.05) (*n* = 9)

^a^Note: A semiquantitative scoring system was used for supranuclear vacuolization (see Materials and Methods for details)

### Correlation Between Tissue Gene Expression and Histopathological Parameters

Pearson’s correlation analyses revealed significant (*p* < 0.05) correlation between many of the histological parameters (Table 6). LP width correlated with SEM width, GCs and SNV; in addition, SEM width correlated with GCs and SNV, EGCs correlated with GCs and SNV, and GCs correlated with SNV (Table 6). Significant correlation was also seen between five of the qPCR analyzed genes and the histological data, four of which correlated with two different histological parameters (Table 7). *Es1* showed significant correlation with SEM width, *epd* correlated with SEM width and SNV (*p* = 0.050), *tmprss9* correlated with LP width and SNV, *GILT* correlated with LP width and SNV, and *mmp13* correlated with GCs and SNV.

**Table 6:** Correlations among histopathological parameters

Histological parameter	Histological parameter	Pearson’s *r* ^a^	*p* value
LP width	SEM width	0.350	0.036
LP width	EGC	0.259	0.127
LP width	GC	0.466	0.004
LP width	SNV	0.479	0.003
SEM width	EGC	0.259	0.127
SEM width	GC	0.400	0.016
SEM width	SNV	0.565	<0.001
EGC	GC	0.600	<0.001
EGC	SNV	0.613	<0.001
GC	SNV	0.652	<0.001

^a^Pearson’s *r* = correlation coefficient

**Table 7:** Correlations among gene expression (RQ data) with histopathological parameters

Gene		LP width	SEM width	EGC	GC	SNV
*pir*	Pearson’s *r* ^a^	−0.059	−0.011	0.036	0.063	0.033
	*p* value	0.733	0.951	0.834	0.716	0.851
*epd*	Pearson’s *r*	0.153	0.364	−0.024	0.207	0.329
	*p* value	0.374	**0.029**	0.888	0.226	**0.050**
*tmprss9*	Pearson’s *r*	0.425	0.224	0.230	0.283	0.456
	*p* value	**0.010**	0.189	0.178	0.095	**0.005**
*napepld*	Pearson’s *r*	−0.058	−0.153	−0.263	−0.216	−0.274
	*p* value	0.736	0.374	0.121	0.205	0.106
*es1*	Pearson’s *r*	0.182	0.355	0.124	0.016	0.255
	*p* value	0.280	**0.034**	0.472	0.928	0.134
*txn*	Pearson’s *r*	−0.079	0.183	−0.011	−0.051	0.151
	*p* value	0.645	0.286	0.948	0.769	0.378
*GILT*	Pearson’s *r*	0.401	0.160	0.047	0.009	0.459
	*p* value	**0.015**	0.351	0.784	0.959	**0.005**
*mmp13*	Pearson’s *r*	−0.061	−0.155	−0.165	−0.413	−0.331
	*p* value	0.722	0.366	0.335	**0.012**	**0.049**
*tgfb*	Pearson’s *r*	−0.114	−0.017	−0.138	−0.300	−0.112
	*p* value	0.509	0.923	0.423	0.075	0.516

The numbers in bold font correspond to *p* value less than or equal to 0.05

^a^Pearson’s *r* = correlation coefficient

## Discussion

### Growth Performance of Salmon

The epithelium of the DI is an important site of nutrient absorption in Atlantic salmon and plays a critical role in growth performance and the maintenance of inflammatory responses against food antigens (Chikwati et al. 2013; Marjara et al. 2012; Moldal et al. 2014; Overland et al. 2009; Sahlmann et al. 2013). As previously reported (Hixson et al. 2016), Atlantic salmon fed CM-containing diets showed a significantly lower weight gain and higher VSI than the fish fed the control diet; however, the CM-fed fish also showed a decreased AFI. This may be a direct result of the inflammation in the intestine, and/or may be due to an aversion of the salmon to the diets containing CM. In this study, the lowest dietary inclusion of CM (8 %) was enough to cause a reduction in growth performance, with similar performance seen in the fish fed the 16CM and 24CM diets (Table 3). Other studies have shown that the inclusion of some PMs (such as lupin meal and wheat gluten) in feeds for Atlantic salmon led to decreased growth performance (Gu et al. 2014), while others showed that some PMs (such as pea meal) could be successfully included up to ~23 % without any apparent detriment to growth performance (Overland et al. 2009). Collectively, these data indicate that Atlantic salmon show different growth performance when fed different PMs. This may be due to the differences in antinutritional factors found in different types of PMs, with some of the main ANFs in CM being glucosinolates (Colombini et al. 2013). PMs from different sources also have different levels of digestibility, which affect the level of nutrient absorption and availability of amino acids needed for growth (Sugiura et al. 1998).

Unlike the current study, Ye et al. (2016) reported no influence of SECM (up to 20 % of the diet) on feed consumption or weight gain of Atlantic salmon. However, Ye et al. (2016) used earlier life stage salmon (parr, ~ 8 g starting weight) compared with the current study (post-smolt, ~240 g starting weight). Collectively, these results suggest that earlier juvenile stages of Atlantic salmon may have a higher tolerance to dietary SECM compared with later juvenile stages.

### The Transcriptional Effect of Dietary CM in the DI

#### Molecular Biomarkers of Chronic Inflammation

The microarray experiment carried out in the current study indicated that the dietary inclusion of 24 % CM resulted in the modulation of the expression of a relatively small number of transcripts related to inflammatory responses, and all of the BLASTX-annotated genes identified by both SAM and RP in the microarray experiment were qPCR validated. The three microarray-identified and qPCR validated genes that were positively correlated with physical signs of inflammation (*tmprss9*, *epd*, and *es1*) are discussed herein to elucidate the transcriptional effect of chronic exposure to dietary CM on inflammatory processes in the DI of Atlantic salmon.

*Tmprss9* was significantly upregulated in the fish fed the 16CM and 24CM diets when compared to fish fed the control diet; this response appeared to be dose-dependent, with the highest degree of upregulation in fish fed the 24CM diet. This gene also showed significant positive correlation with LP width and SNV. *Tmprss9*, also commonly known as *polyserase-I*, was first characterized in Cal et al. (2003). It has been identified as a pro-tumor protease in epithelioid carcinomas (cell line PANC-1) and adenocarcinomas (cell line SK-PC-1) of the pancreas (Fontanil et al. 2014). Cancerous cells demonstrating *tmprss9* expression were found to have significant activation of the urokinase plasminogen activator (uPA) system, which is heavily involved in remodeling of the extracellular matrix (ECM) for the promotion of tumor progression and metastasis (Okumura et al. 2006; Ulisse et al. 2009; Fontanil et al. 2014). This gene and gene product may aid in ECM remodeling to promote a proinflammatory environment in response to the CM-containing diets.

*Epd* was upregulated by all of the CM-containing diets and with no significant difference among CM-fed groups. This gene was positively correlated with SEM width and SNV. *Epd* has been widely studied in the cerebrospinal fluid and central nervous system of teleost fish (Hoffman and Schwarz 1996; Shashoua 1977) and has been linked to neuroplasticity in many species (Pradel et al. 1999; Rother et al. 1995; Shashoua 1991). The physiological role of *epd* outside of the nervous system is largely unknown, but studies have shown that a homolog of this gene is overexpressed during the regeneration of intestinal tissue in the gastrointestinal tract of some invertebrates (Suarez-Castillo et al. 2004; Zheng et al. 2006). There is also evidence that *epd* in teleost fish plays a role in the maintenance of the ECM in response to changing calcium levels (Ganss and Hoffmann 1993). This is significant for the study at hand as the physical signs of inflammation that were seen in the histological analyses are indicative of increased cell turnover and regeneration of damaged intestinal tissue. To our knowledge, this is the first study showing that *epd* is a molecular biomarker of chronic inflammation in the intestine of Atlantic salmon.

*Es1* was significantly upregulated in all fish fed the CM-containing diets with no significant difference between those fed the 8CM, 16CM, or 24CM diets. Additionally, this gene was positively correlated with SEM width. The role of *es1* is not yet understood in humans, but it is proposed that it serves a basic function in the mitochondria (Shin et al. 2004). *Es1* has been shown to be upregulated in the caecum of chicken in response to infection with *Salmonella* (Rychlik et al. 2014), which suggests that *es1* may play a role in the development of immune responses in the vertebrate gut. While the role of *es1* remains largely undetermined, these results, paired with the positive correlation between the gene expression and histology results, collectively suggest it may have an important physiological role in the intestine involved in regulation of inflammatory responses. As with *tmprss9* and *epd*, *es1* appears to be a novel molecular biomarker of chronic inflammation in the salmon intestine.

Two of the qPCR analyzed genes that were not identified by the microarray were also shown to be responsive to CM inclusion. The first gene, *mmp13*, also known as *collagenase-3*, was significantly downregulated by the 24CM diet when compared to the 8CM diet. This gene has been shown to be responsive to numerous inflammatory conditions, including rheumatoid arthritis, wound healing, and inflammatory bowel disease, to name a few (Toriseva et al. 2012; Vizoso et al. 2006). The main function of *mmp13* is in the remodeling of the ECM during proliferation and during events involving high cell turnover, with chronic mucosal inflammation being one of the main conditions in which this gene is responsive (Uitto et al. 1998). The expression profile of *mmp13* (i.e., highest in 8CM-fed fish) and negative correlation of *mmp13* transcript expression with GCs and SNV, were not expected. In Sahlmann et al. (2013), *mmp13* was among the microarray-identified, upregulated differentially expressed genes (DEGs) at day 5 in a 20 % soybean meal (SBM) feeding trial, but was not differentially expressed at any other time points in the 1-week trial. Collectively, these results suggest that *mmp13* expression response to PMs may be dynamic and time-dependent. The potential roles of *mmp13* in PM-associated acute versus chronic inflammation of salmon DI warrant further investigation.

*GILT* transcript expression displayed significant positive correlation with LP width and SNV but did not show any differential expression between the fish fed the different diets. *GILT* has been shown to be heavily involved in many aspects of immunity; it is constitutively expressed in professional antigen presenting cells and can be induced in many other cells by inflammatory cytokines (Phan et al. 2000). GILT aids in the processing of antigenic proteins by reducing disulfide bonds to promote protein unfolding and presentation on MHC proteins for immune recognition (Maric et al. 2001; West and Cresswell 2013). In addition to this, increased *GILT* expression has been linked to decreased T cell activation in mouse models (Barjaktarevic et al. 2006). Although GILT was not significantly differentially expressed between fish fed the different diets, we hypothesize that it may be playing a role in the presentation of food antigens for immune recognition.

The RP analysis revealed four genes with GO terms directly related to “inflammatory response” that were not identified by SAM analysis (ESM, Supplemental Table 1). These genes were *dual oxidase 1* (4.7-fold upregulated), *tumor necrosis factor alpha induced protein 8-like protein 2* (2.4-fold upregulated), *b-cell lymphoma 6 protein homolog* (1.5-fold upregulated), and *c-c motif chemokine 19*, which was represented by two upregulated transcripts (both 2.4-fold upregulated).

Interestingly, RP analysis also identified 20 downregulated immunoglobulin (Ig) chain transcripts (ranging from 1.5-fold to 3.3-fold downregulated). Igs are present in many mucosal surfaces and are necessary for adaptive mucosal immunity (reviewed by Salinas et al. 2011). In fish, the mucosa-associated lymphoid tissue (MALT) contains B cells and various Igs, which are essential for the regulation of response to pathogens in the mucosa (reviewed by Brandtzaeg 2009). The downregulation of Ig encoding transcripts in the 24CM group was surprising, as one might expect Ig transcripts to be upregulated during an inflammatory response. Xue et al. (2015) reported potential immune suppression in Atlantic salmon fed a diet with 100 % replacement of fish oil with camelina oil, solvent-extracted FM, and 10 % CM. This conclusion was based on immune-relevant gene expression results. The downregulation of numerous Ig transcripts may suggest immune suppression in the fish fed the 24CM diet in the current study. Secreted Igs are involved in the maintenance of mucosal immunity (Salinas et al. 2011), and the downregulation of these genes may have influenced the homeostasis of the mucosa in the DI.

#### Other Genes Responsive to Long-Term Dietary CM Exposure

There were some transcripts that were identified by both SAM and RP to be responsive to CM and were qPCR validated, but did not correlate with any of the histological parameters (i.e., *txn*, *napepld*, and *pir*). It is likely that other cellular processes were altered in the CM fed fish, and that these genes may play roles in the modulation of these processes. Those genes are briefly discussed herein to elucidate possible further effects of CM outside of an inflammatory response.

*Txn* was upregulated in all of the fish fed CM-containing diets and there was no significant difference between the fish fed the 8CM, 16CM, or 24CM diets. Txn serves as a key reductase in one of the major reducing pathways in the intracellular environment (Collet and Messens 2010). The thioredoxin system is ubiquitous in biology and plays key roles in resistance to oxidative stress through control of the aggregation of reactive oxygen species (ROS), control of growth, and regulation of apoptosis (Arnér and Holmgren 2000). It is possible that *txn* was upregulated as a protective response to oxidative stress resulting from the long-term dietary exposure to CM.

*Pir* was significantly upregulated in the fish fed all of the CM-containing diets when compared to those fed the control diet but did not correlate with any of the histological parameters. *Pir* is a highly conserved protein found in cell nuclei (Wendler et al. 1997). Its tight association with nuclear factor I has led to suggestions that it may serve as a potential cofactor and could be involved in biological redox reactions through its interaction with iron (Pang et al. 2004; Wendler et al. 1997). It is possible that, as with *txn*, *pir* was upregulated in response to oxidative stress induced by the long-term dietary exposure to CM.

*Napepld* was upregulated in the fish fed the 8CM and 16CM diets when compared to those fed the control diet, but no difference was seen in the fish fed the 24CM diet when compared to the control. *Napepld* encodes a key enzyme in the hydrolysis of many important lipid aldehydes, which generate numerous signaling molecules (Guo et al. 2013). These lipid mediators are involved in myriad cellular responses, including, most notably, innate immunity, energy balance, and response to cellular stress (Magotti et al. 2015). The upregulation of *napepld* in this study may be a result of increased cellular stress because of the CM in the DI.

The gene expression analyses carried out in this study present a snapshot of the Atlantic salmon DI transcriptome response to long-term exposure to dietary CM. The dynamic transcriptome response of salmon intestine to PM-containing diets has been documented in various studies, including Sahlmann et al. (2013), which showed 48 DEGs in response to dietary SBM after 5 days of feeding, but only 5 DEGs after 7 days of feeding. Another study by Sahlmann et al. (2015) showed variable responses to dietary SBM, which peaked at 67 days post-hatching with lower responses from that point onward. This variability was also shown in Marjara et al. (2012), which looked at genomic responses to SBM and demonstrated significant changes in the expression of genes from one time point to another (**i.e.**, from days 3–7 to days 10–21). As shown in the aforementioned studies, the response to dietary PMs is very dynamic and changes significantly over time. In the current study, a high level of variability was also seen between fish, which may have limited our ability to identify DEGs. As a result, it is possible that other cellular processes were altered in response to long-term exposure to dietary CM that we were not able to detect in our analyses.

### Histopathological Changes in the DI in Response to Dietary CM

Transcriptional modulation was paired with histopathological changes in the DI of salmon fed the CM-containing diets that are evidence of the manifestation of an inflammatory response. Quantitative measurements were taken in place of a conventional semi-quantitative scoring system for most histological parameters analyzed to increase the accuracy and sensitivity of the analyses carried out in detecting pathophysiological changes. Notable changes detected include an increase in the population of GCs in the epithelium of fish fed the 16CM and 24CM diets. GCs are responsible for the production and maintenance of the mucus layer that protects the epithelium of the intestine (Kim and Ho 2010). Increased mucus production caused by proliferation of GCs in the intestine is a protective response that can be triggered by food antigens, and an increased population of GCs is a direct indication of this protective response to inflammation (van den Ingh et al. 1991). Increased infiltration of EGCs was also observed in the villi of salmon fed the 24CM diet. EGCs are immune cells found in teleost fish that are functionally homologous to mammalian mast cells (Reite and Evensen 2006). EGCs, which are normally present in relatively high numbers in fish sub-epithelial tissues such as in the digestive tract, gills and skin (Reite 1997; Vergnolle 2004), mediate inflammatory responses through secreting a variety of immuno-active compounds that directly regulate the inflammatory response (Reite 1997). These compounds, which include granular proteases (e.g. serine proteases) and glycoaminoglycans (e.g. heparin) (Reite 1997), are released during degranulation and an increase in resident populations of EGCs has been observed during persistent inflammatory reactions in the intestines of salmonids (Bullock 1963; Sharp et al. 1989). The significant increase in the population of EGCs in the villi of fish fed the 24CM diet suggests that food antigens in the intestinal lumen are eliciting an inflammatory response that is mediated through EGCs.

Other histopathological changes seen in the intestine include a significant thickening of the SEM in fish fed the 24CM diet, which is consistent with the presence of inflammation and has been described in other studies investigating the effect of PMs on the physiology of the DI in salmon (Uran et al. 2008). There was also a widening of the LP seen in the fish fed the 24CM diet. The LP is a layer of connective tissue beneath the epithelium of the intestine and serves as an immune effector site in the intestine (Biorivant et al. 1999; Maxwell 1994). Stimulated T lymphocytes in the LP contribute to the maintenance of proinflammatory processes, and increased T lymphocyte populations are seen in LP widening resulting from inflammatory processes (Circu and Aw 2012). While not directly tested for, our transcriptional evidence suggests the presence of T lymphocytes in the LP through the elevated expression of *thioredoxin*, which is associated with the maintenance of T lymphocyte function (Bennett and Griffiths 2013; Mougiakakos et al. 2011; Sido et al. 2005).

A decrease in SNVs was observed in the fish fed the 16CM and 24CM diets which also provides evidence for an inflammatory response. SNVs are involved in the endocytosis of food nutrients and a decrease in SNVs may be triggered as a protective response against food antigens and ANFs that can cause inflammation (Baeverfjord and Krogdahl 1996). Interestingly, there were no changes observed in the height of the intestinal villi. Typically, inflammation in the DI is associated with shortening of the villi (Baeverfjord and Krogdahl 1996).

Ye (2014) reported similar growth performance results as described in this study: decreased growth performance in the Atlantic salmon smolt fed the high oil residue CM diets with a significant decrease in the final weight and weight gain; the fish fed the CM diets also had significantly lower feed consumption but showed a significantly higher FCR. Ye (2014) also reported a significant increase in GCs in the DI of fish fed the high oil residue CM-containing diets, similar to GC results in the current study (i.e., higher numbers of GCs in fish fed the 16 and 24 % solvent-extracted CM-containing diets). Ye et al. (2016) examined the effect of solvent-extracted CM on the DI of Atlantic salmon parr and reported a significant widening of the LP in response to dietary exposure to 15 % CM- and 20 % CM-containing diets; this study, however, found no significant effect on SNV, GCs or SEM with up to 20 % CM inclusion. Collectively, these results support our previously mentioned hypothesis that Atlantic salmon parr may be more tolerant of CM-containing diets than Atlantic salmon post-smolts.

Many other studies have examined the effects of dietary PMs in the DI of Atlantic salmon. Sahlmann et al. (2013) reported that many immune-relevant transcripts were responsive to SBM inclusion, including *mmp13* and *NFκB*. Sahlmann et al. (2013) also identified several other cellular processes that were responsive to SBM, including lipid metabolism, and cytoskeleton and ECM remodeling. This is similar to what was seen in Marjara et al. (2012), which presented several immune-relevant transcripts that were responsive to SBM. Some notable transcripts include *trypsin, PAR-2A/B*, *interleukin 17A*, and *GILT* (which was found to be non-responsive in the current study). Both of these papers reported histology results that are congruous with what is reported here. Other studies have also reported differential transcript expression and decreased growth performance in Atlantic salmon fed SBM (Skugor et al. 2011; Uran et al. 2008).

Other PMs and plant proteins have been evaluated in Atlantic salmon; decreased growth performance was reported for fish fed narrow leafed lupin and field pea meal (Carter and Hauler 2000), and Tacchi et al. (2012) showed mid-intestine transcriptional changes in response to dietary plant proteins. Overland et al. (2009), however, showed no significant change in growth performance in fish fed SBM or pea protein concentrate when compared to a FM diet.

## Conclusions

The data presented in the current study demonstrate an inflammatory reaction in response to long-term dietary CM exposure in the DI of Atlantic salmon post-smolts. This conclusion was established based on decreased overall growth performance in response to dietary CM inclusion, which was paired with the modulation of the expression of some novel inflammation-relevant genes and histopathological changes associated with inflammation (i.e., LP and SEM widening, increased CGs and EGCs, and decreased SNV). Some of the identified genes correlated significantly with histopathological parameters and allowed for the identification of novel molecular biomarkers of inflammation (i.e., *tmprss9, epd*, and *es1*) that are responsive to long-term dietary CM exposure. While high levels of biological variability may have limited out ability to detect all of the genes that were responding to the CM-containing diets, we believe that this study provides valuable new information for understanding the long-term effects of dietary CM exposure in the DI of Atlantic salmon.

## Electronic Supplementary Material

Below is the link to the electronic supplementary material.

Supplemental Table 1 (DOCX 26 kb)

Supplemental Table 2 (DOCX 20 kb)

## References

[CR1] Acamovic T, Gilbert C, Lamb K, Walker KC (1999). Nutritive value of *Camelina sativa* meal for poultry. Br Poult Sci.

[CR2] Amarowicz R, Estrella I, Hernandez T, Robredo S, Troszynska A, Kosinska A, Pegg RB (2010). Free radical-scavenging capacity, antioxidant activity, and phenolic composition of green lentils (*Lens culinaris*). Food Chem.

[CR3] Arnér ES, Holmgren A (2000) Physiological functions of thioredoxin and thioredoxin reductase. Eur J Biochem 267:6102–6109 10.1046/j.1432-1327.2000.01701.x 11012661

[CR4] Baeverfjord G, Krogdahl A (1996). Development and regression of soybean meal induced enteritis in Atlantic salmon, *Salmo salar* L., distal intestine: a comparison with the intestine of fasted fish. J Fish Dis.

[CR5] Barjaktarevic I, Rahman A, Radoja S, Bogunovic B, Vollmer A, Vukmanovic S, Maric M (2006). Inhibitory role of IFN-gamma-inducible lysosomal thiol reductase in T cell activation. J Immunol.

[CR6] Bennett SJ, Griffiths HR, Alcaraz MJ, Gualillo O, Sánchez-Pernaute O (2013). Regulation of T-cell functions by oxidative stress. Oxidative stress in applied basic research and clinical practice.

[CR7] Biorivant M, Marini M, Di Felice G, Pronio AM, Montesani C, Tersigni R, Strober W (1999). Lamina propria T cells in Crohn’s disease and other gastrointestinal inflammation show defective CD2 pathway-induced apoptosis. Gastroenterology.

[CR8] Bo TH, Dysvik B, Jonassen I (2004). LSimpute: accurate estimation of missing values in microarray data with least squares methods. Nucleic Acids Res.

[CR9] Booman M, Borza T, Feng CY, Hori TS, Higgins B, Culf A, Leger D, Chute IC, Belkaid A, Rise M, Gamperl AK, Hubert S, Kimball J, Ouellette RJ, Johnson SC, Bowman S, Rise ML (2011). Development and experimental validation of a 20K Atlantic cod (*Gadus morhua*) oligonucleotide microarray based on a collection of over 150,000 ESTs. Mar Biotechnol.

[CR10] Brandtzaeg P (2009). Mucosal immunity: induction, dissemination, and effector functions. Scand J Immunol.

[CR11] Breitling R, Armengaud P, Amtmann A, Herzyk P (2004). Rank products: a simple, yet powerful, new method to detect differentially regulated genes in replicated microarray experiments. FEBS Lett.

[CR12] Bullock WL (1963). Intestinal histology of some salmonid fishes with particular reference to the histopathology of acanthocephalan infections. J Morphol.

[CR13] Cal S, Quesada V, Garabaya C, Lopez-Otin C (2003). Polyserase-I, a human polyprotease with the ability to generate independent serine protease domains from a single translation product. Proc Natl Acad Sci U S A.

[CR14] Carter CG, Hauler RC (2000). Fish meal replacement by plant meals in extruded feeds for Atlantic salmon, *Salmo salar* L. Aquaculture.

[CR15] Cherian G, Makkar HPS (2012). *Camelina sativa* in poultry diets: oppotunities and challenges. Biofuel co-products as livestock feed: opportunities and challenges.

[CR16] Chikwati EM, Sahlmann C, Holm H, Penn MH, Krogdahl A, Bakke AM (2013). Alterations in digestive enzyme activities during the development of diet-induced enteritis in Atlantic salmon, *Salmo salar* L. Aquaculture.

[CR17] Chiu CC, Chan SY, Wang CC, Wu WS (2013). Missing value imputation for microarray data: a comprehensive comparison study and a web tool. BMC Syst Biol.

[CR18] Circu ML, Aw TY (2012). Intestinal redox biology and oxidative stress. Semin Cell Dev Biol.

[CR19] Collet JF, Messens J (2010). Structure, function, and mechanism of thioredoxin proteins. Antioxid Redox Signal.

[CR20] Colombini S, Broderick GA, Galasso I, Martinello T, Rapetti L, Russo R, Reggiani R (2013). Evaluation of *Camelina sativa* (L.) Crantz meal as an alternative protein source in ruminant rations. J Sci Food Agric.

[CR21] Conesa A, Götz S, García-Gómez JM, Terol J, Talón M, Robles M (2005). Blast2GO: a universal tool for annotation, visualization and analysis in functional genomics research. Bioinformatics.

[CR22] FAO (2009). The state of world fisheries and aquaculture 2008.

[CR23] FAO (2012) Fisheries and aquaculture statistics. Food and Aquaculture Organization of the United Nations, Rome

[CR24] Fontanil T, Mohamedi Y, Esteban MM, Obaya AJ, Cal S (2014). Polyserase-1/TMPRSS9 induces pro-tumor effects in pancreatic cancer cells by activation of pro-uPA. Oncol Rep.

[CR25] Frame DD, Palmer M, Peterson B (2007). Use of *Camelina sativa* in the diets of young turkeys. J Appl Poult Res.

[CR26] Ganss B, Hoffmann W (1993). Calcium binding to sialic acids and its effect on the conformation of ependymins. Eur J Biochem.

[CR27] Ghamkhar K, Croser J, Aryamanesh N, Campbell M, Kon’kova N, Francis C (2010). Camelina (*Camelina sativa* (L.) Crantz) as an alternative oilseed: molecular and ecogeographic analyses. Genome.

[CR28] Gu M, Kortner TM, Penn M, Hansen AK, Krogdahl A (2014). Effects of dietary plant meal and soya-saponin supplementation on intestinal and hepatic lipid droplet accumulation and lipoprotein and sterol metabolism in Atlantic salmon (*Salmo salar* L.). Br J Nutr.

[CR29] Guo L, Gragg SD, Chen Z, Zhang Y, Amarnath V, Davies SS (2013). Isolevuglandin-modified phosphatidylethanolamine is metabolized by NAPE-hydrolyzing phospholipase D. J Lipid Res.

[CR30] Hixson SM, Parrish CC, Anderson DM (2014). Full substitution of fish oil with camelina (*Camelina sativa*) oil, with partial substitution of fish meal with camelina meal, in diets for farmed Atlantic salmon (*Salmo salar*) and its effect on tissue lipids and sensory quality. Food Chem.

[CR31] Hixson SM, Parrish CC, Wells JS, Winkowski EM, Anderson DM, Bullerwell CN (2016). Inclusion of camelina meal as a protein source in diets for farmed salmonids. Aquac Nutr.

[CR32] Hoffman W, Schwarz H (1996). Ependymins: meningeal-derived extracellular matrix proteins at the blood–brain barrier. Int Rev Cytol.

[CR33] Hong F, Breitling R, McEntee CW, Wittner BS, Nemhauser JL, Chory J (2006). RankProd: a bioconductor package for detecting differentially expressed genes in meta-analysis. Bioinformatics.

[CR34] Jantzen SG, Sanderson DS, Von Schalburg KR, Yasuike M, Marass F, Koop BF (2011). A 44K microarray dataset of the changing transcriptome in developing Atlantic salmon (*Salmo salar* L.). BMC Res Notes.

[CR35] Jeffery IB, Desmond GH, Culhane AC (2006). Comparison and evaluation of methods for generating differentially expressed gene lists from microarray data. BMC Bioinformatics.

[CR36] Kim YS, Ho SB (2010). Intestinal goblet cells and mucins in health and disease: recent insights and progress. Curr Gastroenterol Rep.

[CR37] Livak KJ, Schmittgen TD (2001). Analysis of relative gene expression data using real-time quantitative PCR and the 2(−delta delta C(T)) method. Methods.

[CR38] Lokka G, Austbo L, Falk K, Bjerkas I, Koppang EO (2013). Intestinal morphology of the wild Atlantic salmon (*Salmo salar*). J Morphol.

[CR39] Magotti P, Bauer I, Igarashi M, Babagoli M, Marotta R, Piomelli D, Garau G (2015). Structure of human N-acylphosphatidylethanolamine-hydrolyzing phospholipase D: regulation of fatty acid ethanolamide biosynthesis by bile acids. Structure.

[CR40] Maric M, Arunachalam B, Phan UT, Dong C, Garrett WS, Cannon KS, Alfonso C, Karlsson L, Flavell RA, Cresswell P (2001). Defective antigen processing in GILT-free mice. Science.

[CR41] Marjara IS, Chikwati EM, Valen EC, Krogdahl A, Bakke AM (2012). Transcriptional regulation of IL-17A and other inflammatory markers during the development of soybean meal-induced enteropathy in the distal intestine of Atlantic salmon (*Salmo salar* L.). Cytokine.

[CR42] Maxwell WL (1994). Wheater’s functional histology.

[CR43] Moldal T, Løkka G, Wiik-Nielsen J, Austbø L, Torstensen BE, Rosenlund G, Dale OB, Kaldhusdal M, Koppang OA (2014). Substitution of dietary fish oil with plant oils is associated with shortened mid intestinal folds in Atlantic salmon (*Salmo salar*). BMC Vet Res.

[CR44] Mougiakakos D, Johansson CC, Jitschin R, Bottcher M, Kiessling R (2011). Increased thioredoxin-1 production in human naturally occurring regulatory T cells confers enhanced tolerance to oxidative stress. Blood.

[CR45] National Research Council (2011). Nutrient requirements of fish and shrimp.

[CR46] Okumura Y, Hayama M, Takahashi E, Fujiuchi M, Shimabukuro A, Yano M, Kido H (2006). Serase-1B, a new splice variant of polyserase-1/TMPRSS9, activates urokinase-type plasminogen activator and the proteolytic activation is negatively regulated by glycosaminoglycans. Biochem J.

[CR47] Overland M, Sorensen M, Storebakken T, Penn M, Krogdahl A, Skrede A (2009). Pea protein concentrate substituting fish meal or soybean meal in diets for Atlantic salmon (*Salmo salar*)—effect on growth performance, nutrient digestibility, carcass composition, gut health, and physical feed quality. Aquaculture.

[CR48] Pang H, Bartlam M, Zeng Q, Miyatake H, Hisano T, Miki K, Wong LL, Gao GF, Rao Z (2004). Crystal structure of human pirin: an iron-binding nuclear protein and transcription cofactor. J Biol Chem.

[CR49] Pfaffl MW (2001). A new mathematical model for relative quantification in real-time RT-PCR. Nucleic Acids Res.

[CR50] Phan UT, Arunachalam B, Cresswell P (2000). Gamma-interferon-inducible lysosomal thiol reductase (GILT). Maturation, activity, and mechanism of action. J Biol Chem.

[CR51] Pradel G, Schachner M, Schmidt R (1999). Inhibition of memory consolidation by antibodies against cell adhesion molecules after active avoidance conditioning in zebrafish. J Neurobiol.

[CR52] Reite OB (1997). Mast cells/eosinophilic granule cells of salmonids: staining properties and responses to noxious agents. Fish Shellfish Immunol.

[CR53] Reite OB, Evensen O (2006). Inflammatory cells of teleostean fish: a review focusing on mast cells/eosinophilic granule cells and rodlet cells. Fish Shellfish Immunol.

[CR54] Rother S, Schmidt R, Brysch W, Schlingensiepen KH (1995). Learning-induced expression of meningeal ependymin mRNA and demonstration of ependymin in neurons and glial cells. J Neurochem.

[CR55] Russo R, Reggiani R (2012). Antinutritive compounds in twelve *Camelina sativa* genotypes. Am J Plant Sci.

[CR56] Rychlik I, Elsheimer-Matulova M, Kyrova K (2014). Gene expression in the chicken caecum in response to infections with non-typhoid Salmonella. Vet Res.

[CR57] Sahlmann C, Sutherland BJ, Kortner TM, Koop BF, Krogdahl A, Bakke AM (2013). Early response of gene expression in the distal intestine of Atlantic salmon (*Salmo salar* L.) during the development of soybean meal induced enteritis. Fish Shellfish Immunol.

[CR58] Sahlmann C, Gu J, Kortner TM, Lein I, Krogdahl A, Bakke AM (2015). Ontogeny of the digestive system of Atlantic salmon (*Salmo salar* L.) and effects of soybean meal from start-feeding. PLoS One.

[CR59] Salinas I, Zhang Y-A, Sunyer LO (2011). Mucosal immunoglobulins and B cells of teleost fish. Dev Comp Immunol.

[CR60] Schlemmer U, Frolich W, Prieto RM, Grases F (2009). Phytate in foods and significance for humans: food sources, intake, processing, bioavailability, protective role and analysis. Mol Nutr Food Res.

[CR61] Schneider CA, Rasband WS, Eliceiri KW (2012). NIH Image to ImageJ: 25 years of image analysis. Nat Methods.

[CR62] Sharp GJE, Pike AW, Secombes CJ (1989). The immune response of wild rainbow trout, *Salmo gairdneri* Richardson, to naturally acquired plerocercoid infections of *Diphyllobothrium dendriticum* (Nitzsch, 1824) and *D. ditremum* (Creplin, 1825). J Fish Biol.

[CR63] Shashoua VE (1977). Brain protein metabolism and the acquisition of new patterns of behavior. Proc Natl Acad Sci U S A.

[CR64] Shashoua VE (1991). Ependymin, a brain extracellular glycoprotein, and CNS plasticity. Ann N Y Acad Sci.

[CR65] Shin JH, Weitzdoerfer R, Fountoulakis M, Lubec G (2004). Expression of cystathionine beta-synthase, pyridoxal kinase, and ES1 protein homolog (mitochondrial precursor) in fetal Down syndrome brain. Neurochem Int.

[CR66] Sido B, Giese T, Autschbach F, Lasitschka F, Braunstein J, Meuer SC (2005). Potential role of thioredoxin in immune responses in intestinal lamina propria T lymphocytes. Eur J Immunol.

[CR67] Sire MF, Lutton C, Vernier JM (1981). New views on intestinal absorption of lipids in teleostean fishes: an ultrastructural and biochemical study in the rainbow trout. J Lipid Res.

[CR68] Skugor S, Grisdale-Helland B, Afanasyev S, Vielma J, Krasnov A (2011). Gene expression responses to restricted feeding and extracted soybean meal in Atlantic salmon (*Salmo salar* L.). Aquac Nutr.

[CR69] Suarez-Castillo EC, Medina-Ortiz WE, Roig-Lopez JL, Garcia-Arraras J (2004). Ependymin, a gene involved in regeneration and neuroplasticity in vertebrates, is overexpressed during regeneration in the echinoderm *Holothuria glaberrima*. Gene.

[CR70] Sugiura SH, Dong FM, Rathbone CK, Hardy RW (1998). Apparent protein digestibility and mineral availabilities in various feed ingredients for salmonid feeds. Aquaculture.

[CR71] Tacchi L, Secombes CJ, Bickerdike R, Adler MA, Venegas C, Takle H, Martin SA (2012). Transcriptomic and physiological responses to fishmeal substitution with plant proteins in formulated feed in farmed Atlantic salmon (*Salmo salar*). BMC Genomics.

[CR72] Tacon AJ, Metian M (2008). Global overview on the use of fish meal and fish oil in industrially compounded aquafeeds: Trends and future prospects. Aquaculture.

[CR73] Tacon AJ, Metian MM (2009). Fishing for aquaculture: non-food use of small pelagic forage fish—a global perspective. Rev Fish Sci.

[CR74] Tacon AJ, Metian M, Turchini GM, De Silva SS (2009). Responsible aquaculture and trophic level implications to global fish supply. Rev Fish Sci.

[CR75] Toriseva M, Laato M, Carpen O, Ruohonen ST, Savontaus E, Inada M, Krane SM, Kahari VM (2012). MMP-13 regulates growth of wound granulation tissue and modulates gene expression signatures involved in inflammation, proteolysis, and cell viability. PLoS One.

[CR76] Tusher VG, Tibshirani R, Chu G (2001). Significance analysis of microarrays applied to the ionizing radiation response. Proc Natl Acad Sci U S A.

[CR77] Uitto VJ, Airola K, Vaalamo M, Johansson N, Putnins EE, Firth JD, Salonen J, Lopez-Otin C, Saarialho-Kere U, Kahari VM (1998). Collagenase-3 (matrix metalloproteinase-13) expression is induced in oral mucosal epithelium during chronic inflammation. Am J Pathol.

[CR78] Ulisse S, Baldini E, Sorrenti S, D’armiento M (2009). The urokinase plasminogen activator system: a target for anti-cancer therapy. Curr Cancer Drug Targets.

[CR79] Uran PA, Schrama JW, Rombout JHWM, Obach A, Jensen L, Koppe W, Verreth JAJ (2008). Soybean meal-induced enteritis in Atlantic salmon (*Salmo salar* L.) at different temperatures. Aquac Nutr.

[CR80] Van Den Ingh TSGAM, Krogdahl A, Olli JJ, Hendriks HGCJM, Koninkx JGJF (1991). Effects of soybean-containing diets on the proximal and distal intestine in Atlantic salmon (*Salmo salar*): a morphological study. Aquaculture.

[CR81] Vandesompele J, De Preter K, Pattyn F, Poppe B, Van Roy N, De Paepe A, Speleman F (2002). Accurate normalization of real-time quantitative RT-PCR data by geometric averaging of multiple internal control genes. Genome Biol.

[CR82] Vergnolle N (2004). Modulation of visceral pain and inflammation by protease-activated receptors. Br J Pharmacol.

[CR83] Vizoso FJ, Gonzalez LO, Corte MD, Corte MG, Bongera M, Martinez A, Martin A, Andicoechea A, Gava RR (2006). Collagenase-3 (MMP-13) expression by inflamed mucosa in inflammatory bowel disease. Scand J Gastroenterol.

[CR84] Wendler WM, Kremmer E, Forster R, Winnacker EL (1997). Identification of pirin, a novel highly conserved nuclear protein. J Biol Chem.

[CR85] West LC, Cresswell P (2013). Expanding roles for GILT in immunity. Curr Opin Immunol.

[CR86] Xue X, Hixson SM, Hori TS, Booman M, Parrish CC, Anderson DM, Rise ML (2015). Atlantic salmon (*Salmo salar*) liver transcriptome response to diets containing *Camelina sativa* products. Comp Biochem Physiol Part D Genomics Proteomics.

[CR87] Ye C (2014) Evaluation of camelina (*Camelina sativa*) byproducts fed to Atlantic salmon (*Salmo salar*) in practical diets. M.Sc. Thesis. Dalhousie University, Truro, NS, Canada

[CR88] Ye C, Anderson DM, Lall SP (2016). The effects of camelina oil and solvent extracted camelina meal on the growth, carcass composition and hindgut histology of Atlantic salmon (*Salmo salar*) parr in freshwater. Aquaculture.

[CR89] Zheng FX, Sun XQ, Fang BH, Hong XG, Zhang JX (2006). Comparative analysis of genes expressed in regenerating intestine and non-eviscerated intestine of *Apostichopus japonicus* Selenka (Aspidochirotida: Stichopodidae) and cloning of ependymin gene. Hydrobiologia.

[CR90] Zubr J (2003). Dietary fatty acids and amino acids of *Camelina sativa* seed. J Food Qual.

