# Supplementary material for: Functional Genomic Analysis of the Impact of Camelina (Camelina sativa) Meal on Atlantic Salmon (Salmo salar) Distal Intestine Gene Expression and Physiology
Source: Mar Biotechnol (NY). 2016 Jun 2;18:418–35. doi: 10.1007/s10126-016-9704-x (PMC4911373; doi:10.1007/s10126-016-9704-x)
Supplement: Supplementary file 1 — (DOCX 26 kb) [file 10126_2016_9704_MOESM1_ESM.docx]

Supplemental Table 1. The rank products (RP) method identified 82 features that were significantly up-regulated in the distal intestine of salmon fed the 24CM diet compared to salmon fed control diet (percentage of false-positives 5%).

| **Probe ID^1^** | **Fold change^2^** | **Best named BLASTx hit^3^** | **Gene Ontology^4^** |
| --- | --- | --- | --- |
| C175R008 | 4.79 | Fucolectin-4 | F:binding |
| C027R089 | 4.70 | Dual oxidase 1 | C:integral component of membrane; C:extracellular exosome; F:peroxidase activity; F:calcium ion binding; F:NAD(P)H oxidase activity; F:heme binding; P:leukocyte migration involved in inflammatory response; P:thyroid hormone generation; P:response to oxidative stress; P:fertilization; P:response to wounding; P:response to virus; P:response to bacterium; P:bone mineralization; P:thyroid gland development; P:multicellular organism growth; P:macrophage chemotaxis; P:inner ear development; P:adenohypophysis morphogenesis; P:hydrogen peroxide biosynthetic process; P:defense response to fungus; P:oxidation-reduction process |
| C132R013 | 4.11 | 26S protease regulatory subunit 4 | C:nucleoplasm; C:proteasome regulatory particle, base subcomplex; C:integral component of membrane; C:cell junction; C:nuclear proteasome complex; C:cytosolic proteasome complex; F:potassium channel activity; F:ATP binding; F:peptidase activity; F:TBP-class protein binding; F:proteasome-activating ATPase activity; F:poly(A) RNA binding; P:mitotic S phase; P:mitotic anaphase; P:activation of MAPKK activity; P:protein polyubiquitination; P:stimulatory C-type lectin receptor signaling pathway; P:antigen processing and presentation of exogenous peptide antigen via MHC class I, TAP-dependent; P:regulation of cellular amino acid metabolic process; P:DNA damage response, signal transduction by p53 class mediator resulting in cell cycle arrest; P:epidermal growth factor receptor signaling pathway; P:Ras protein signal transduction; P:axon guidance; P:insulin receptor signaling pathway; P:fibroblast growth factor receptor signaling pathway; P:viral process; P:ER-associated ubiquitin-dependent protein catabolic process; P:anaphase-promoting complex-dependent proteasomal ubiquitin-dependent protein catabolic process; P:tumor necrosis factor-mediated signaling pathway; P:NIK/NF-kappaB signaling; P:Fc-epsilon receptor signaling pathway; P:negative regulation of apoptotic process; P:positive regulation of RNA polymerase II transcriptional preinitiation complex assembly; P:vascular endothelial growth factor receptor signaling pathway; P:neurotrophin TRK receptor signaling pathway; P:negative regulation of ubiquitin-protein ligase activity involved in mitotic cell cycle; P:positive regulation of ubiquitin-protein ligase activity involved in regulation of mitotic cell cycle transition; P:potassium ion transmembrane transport; P:negative regulation of canonical Wnt signaling pathway; P:positive regulation of canonical Wnt signaling pathway; P:positive regulation of proteasomal protein catabolic process |
| **C202R142** | **3.87** | **---NA---** | **-** |
| C058R023 | 3.85 | ---NA--- | - |
| **C251R109** | **3.79** | **Pirin** | **C:nucleus; C:cytoplasm; F:transcription cofactor activity; F:quercetin 2,3-dioxygenase activity; F:metal ion binding; P:monocyte differentiation; P:oxidation-reduction process; P:regulation of nucleic acid-templated transcription** |
| C208R110 | 3.78 | ---NA--- | - |
| C022R164 | 3.67 | Cocaine- and amphetamine-regulated transcript precursor | C:extracellular space; C:cell; P:activation of MAPKK activity; P:cellular glucose homeostasis; P:G-protein coupled receptor signaling pathway; P:adult feeding behavior; P:cellular response to starvation; P:negative regulation of appetite |
| **C011R096** | **3.52** | **PREDICTED: ES1 protein homolog, mitochondrial-like** | **-** |
| C134R154 | 3.46 | Acyl-CoA-binding protein | C:mitochondrion; C:extracellular exosome; C:perinuclear endoplasmic reticulum; F:carbohydrate binding; F:long-chain fatty acyl-CoA binding; F:protein dimerization activity; P:behavioral fear response; P:triglyceride metabolic process; P:learning or memory; P:lateral ventricle development; P:phosphatidylcholine acyl-chain remodeling; P:skin development; P:long-term synaptic potentiation |
| C079R095 | 3.15 | Selenoprotein Ja | - |
| C147R042 | 3.13 | PREDICTED: ES1 protein homolog, mitochondrial-like | - |
| C162R055 | 3.07 | Nucleoside diphosphate kinase 3 | C:mitochondrion; C:cytosol; C:extracellular exosome; F:nucleoside diphosphate kinase activity; F:ATP binding; F:metal ion binding; P:nucleoside diphosphate phosphorylation; P:GTP biosynthetic process; P:UTP biosynthetic process; P:CTP biosynthetic process; P:regulation of apoptotic process |
| C097R069 | 3.00 | MHC class I heavy chain | C:integral component of membrane; C:MHC class I protein complex; F:protein binding; F:peptide antigen binding; P:antigen processing and presentation of peptide antigen via MHC class I; P:immune response |
| C222R099 | 2.93 | C-type mannose binding lectin precursor | C:extracellular space; C:cytosol; C:plasma membrane; C:brush border; C:cytolytic granule; F:chitin binding; F:carbohydrate binding |
| C170R062 | 2.92 | PREDICTED: chromodomain-helicase-DNA-binding protein 4-like isoform X5 | C:NuRD complex; F:DNA binding; F:ATP binding; F:microtubule binding; F:ATP-dependent helicase activity; F:zinc ion binding; P:chromatin organization; P:regulation of transcription, DNA-templated; P:fin regeneration; P:spindle assembly |
| C215R136 | 2.86 | Alpha-2,8-sialyltransferase 8F | C:Golgi apparatus; C:integral component of membrane; F:sialyltransferase activity; P:ganglioside biosynthetic process; P:protein O-linked glycosylation; P:oligosaccharide metabolic process; P:carbohydrate biosynthetic process; P:sialylation |
| C173R048 | 2.85 | PREDICTED: fatty acid synthase-like, partial | F:transferase activity; P:metabolic process |
| C071R101 | 2.84 | Phosphoenolpyruvate carboxykinase, mitochondrial precursor | C:mitochondrion; C:extracellular exosome; F:phosphoenolpyruvate carboxykinase (ATP) activity; F:phosphoenolpyruvate carboxykinase (GTP) activity; F:GTP binding; F:kinase activity; P:gluconeogenesis; P:response to glucose; P:phosphorylation |
| C109R055 | 2.81 | Deoxyribonuclease gamma precursor | F:deoxyribonuclease activity; P:DNA catabolic process |
| C178R063 | 2.77 | PREDICTED: allantoinase, mitochondrial-like isoform X1 | F:allantoinase activity; F:zinc ion binding; F:cobalt ion binding; P:allantoin catabolic process |
| **C231R006** | **2.74** | **N-acyl-phosphatidylethanolamine-hydrolyzing phospholipase D** | **C:cytoplasm; C:photoreceptor outer segment membrane; C:extracellular exosome; F:zinc ion binding; F:N-acylphosphatidylethanolamine-specific phospholipase D activity; P:retinoid metabolic process; P:phototransduction, visible light; P:phospholipid catabolic process** |
| C037R103 | 2.72 | PREDICTED: protein phosphatase 1 regulatory subunit 27 | F:phosphatase binding; P:negative regulation of phosphatase activity |
| C187R161 | 2.62 | PREDICTED: transcription activator BRG1 isoform X1 | C:SWI/SNF complex; F:ATP binding; F:ATPase activity; F:histone binding; P:activation of MAPKKK activity; P:heart looping; P:retinal pigment epithelium development; P:regulation of transcription, DNA-templated; P:central nervous system development; P:retina layer formation; P:neural crest cell development; P:optic nerve formation; P:neuron differentiation; P:fin regeneration; P:ATP-dependent chromatin remodeling; P:embryonic camera-type eye morphogenesis; P:cardiac muscle cell proliferation; P:embryonic retina morphogenesis in camera-type eye; P:cell migration involved in heart development |
| C236R052 | 2.60 | Thioredoxin | C:extracellular region; C:nucleus; C:mitochondrion; F:binding; F:protein disulfide oxidoreductase activity; F:oxidoreductase activity, acting on a sulfur group of donors, disulfide as acceptor; P:sulfate assimilation; P:positive regulation of protein phosphorylation; P:nucleobase-containing compound metabolic process; P:protein folding; P:glycerol ether metabolic process; P:cell communication; P:gene expression; P:regulation of intracellular protein transport; P:cellular response to oxidative stress; P:positive regulation of molecular function; P:single organism signaling; P:cell redox homeostasis; P:regulation of nucleocytoplasmic transport; P:negative regulation of cellular process; P:regulation of response to stimulus; P:oxidation-reduction process |
| **C215R002** | **2.59** | **PREDICTED: transmembrane protease serine 9-like** | **F:serine-type endopeptidase activity; P:proteolysis** |
| C091R122 | 2.57 | PREDICTED: keratin-associated protein 10-4-like | P:lipid metabolic process; F:calcium ion binding; P:metabolic process; P:microtubule cytoskeleton organization; F:microtubule binding; C:microtubule; C:membrane; P:antigen processing and presentation; P:immune response |
| C027R010 | 2.57 | Protein transport protein Sec23A | C:Golgi membrane; C:cytosol; C:COPII vesicle coat; C:smooth endoplasmic reticulum membrane; C:perinuclear region of cytoplasm; F:protein binding; F:zinc ion binding; P:antigen processing and presentation of peptide antigen via MHC class I; P:intracellular protein transport; P:protein N-linked glycosylation via asparagine; P:antigen processing and presentation of exogenous peptide antigen via MHC class II; P:pectoral fin morphogenesis; P:post-translational protein modification; P:small molecule metabolic process; P:COPII vesicle coating; P:embryonic neurocranium morphogenesis; P:cartilage development |
| C086R064 | 2.53 | PREDICTED: gap junction Cx32.2 protein-like | C:connexon complex; C:integral component of membrane; P:cell communication |
| C146R083 | 2.52 | PREDICTED: lysophospholipid acyltransferase 7 | C:endoplasmic reticulum membrane; C:integral component of membrane; F:dihydrolipoamide branched chain acyltransferase activity; F:protein binding; F:palmitoleoyl [acyl-carrier-protein]-dependent acyltransferase activity; F:carnitine O-acyltransferase activity; F:acylglycerol O-acyltransferase activity; F:serine O-acyltransferase activity; F:O-acetyltransferase activity; F:O-octanoyltransferase activity; F:O-palmitoyltransferase activity; F:S-acetyltransferase activity; F:S-malonyltransferase activity; F:C-palmitoyltransferase activity; F:N-succinyltransferase activity; F:O-succinyltransferase activity; F:S-succinyltransferase activity; F:O-sinapoyltransferase activity; F:peptidyl-lysine N6-myristoyltransferase activity; F:peptidyl-lysine N6-palmitoyltransferase activity; F:benzoyl acetate-CoA thiolase activity; F:3-hydroxybutyryl-CoA thiolase activity; F:3-ketopimelyl-CoA thiolase activity; F:acyl-CoA N-acyltransferase activity; F:protein-cysteine S-myristoyltransferase activity; F:glucosaminyl-phosphotidylinositol O-acyltransferase activity; F:ergosterol O-acyltransferase activity; F:lanosterol O-acyltransferase activity; F:naphthyl-2-oxomethyl-succinyl-CoA succinyl transferase activity; F:2,4,4-trimethyl-3-oxopentanoyl-CoA 2-C-propanoyl transferase activity; F:2-methylhexanoyl-CoA C-acetyltransferase activity; F:butyryl-CoA 2-C-propionyltransferase activity; F:2,6-dimethyl-5-methylene-3-oxo-heptanoyl-CoA C-acetyltransferase activity; F:L-2-aminoadipate N-acetyltransferase activity; F:UDP-3-O-[3-hydroxymyristoyl] glucosamine N-acyltransferase activity; F:keto acid formate lyase activity; F:Ras palmitoyltransferase activity; F:azetidine-2-carboxylic acid acetyltransferase activity; F:peptidyl-lysine N-acetyltransferase activity, acting on acetyl phosphate as donor; F:acetyl-CoA:L-lysine N6-acetyltransferase; P:brain development; P:phosphatidylinositol acyl-chain remodeling; P:small molecule metabolic process; P:glycerophospholipid biosynthetic process |
| C060R142 | 2.52 | PREDICTED: small ubiquitin-related modifier 1-B-like | F:protein binding |
| C066R045 | 2.51 | Carboxypeptidase A1 precursor | C:extracellular space; F:metallocarboxypeptidase activity; F:zinc ion binding; P:proteolysis |
| C238R071 | 2.50 | ---NA--- | - |
| C240R068 | 2.44 | C-C motif chemokine 19 | C:extracellular region; F:chemokine activity; P:immune response; P:dorsal/ventral pattern formation; P:leukocyte chemotaxis; P:regulation of cellular process; P:cellular response to cytokine stimulus |
| C104R012 | 2.43 | Duodenase-1 precursor | F:serine-type endopeptidase activity; P:proteolysis |
| C120R011 | 2.42 | C1 inhibitor | C:extracellular exosome; C:blood microparticle; F:serine-type endopeptidase inhibitor activity; F:protein binding; P:negative regulation of complement activation, lectin pathway; P:negative regulation of endopeptidase activity; P:regulation of blood coagulation; P:single-organism cellular process |
| **C227R083** | **2.40** | **Thioredoxin** | **C:extracellular region; C:nucleus; C:mitochondrion; F:protein disulfide oxidoreductase activity; F:oxidoreductase activity, acting on a sulfur group of donors, disulfide as acceptor; P:sulfate assimilation; P:regulation of transcription, DNA-templated; P:protein folding; P:glycerol ether metabolic process; P:response to radiation; P:regulation of protein import into nucleus, translocation; P:cellular response to oxidative stress; P:B cell proliferation; P:positive regulation of DNA binding; P:cell redox homeostasis; P:oxidation-reduction process** |
| C246R138 | 2.40 | Tumor-associated calcium signal transducer 2 precursor | C:basolateral plasma membrane; C:perinuclear region of cytoplasm; P:liver development; P:single organismal cell-cell adhesion; P:negative regulation of cellular process; P:epiboly involved in gastrulation with mouth forming second |
| C024R134 | 2.39 | C-C motif chemokine 13 precursor | C:extracellular region; F:protein kinase activity; F:ATP binding; F:chemokine activity; F:CCR1 chemokine receptor binding; P:protein phosphorylation; P:immune response; P:cytoskeleton organization; P:signal transduction; P:regulation of cell shape; P:eosinophil chemotaxis; P:cellular response to cytokine stimulus; P:positive regulation of natural killer cell chemotaxis |
| C137R126 | 2.39 | C-C motif chemokine 19 | C:extracellular space; F:chemokine activity; F:CCR7 chemokine receptor binding; P:monocyte chemotaxis; P:inflammatory response; P:G-protein coupled receptor signaling pathway; P:dorsal/ventral pattern formation; P:neutrophil chemotaxis; P:regulation of localization; P:positive regulation of GTPase activity; P:lymphocyte chemotaxis; P:chemokine-mediated signaling pathway; P:positive regulation of ERK1 and ERK2 cascade; P:cellular response to interferon-gamma; P:cellular response to interleukin-1; P:cellular response to tumor necrosis factor |
| C168R011 | 2.36 | Coiled-coil transcriptional coactivator b | - |
| C126R076 | 2.36 | PREDICTED: thrombospondin-4-like | C:basement membrane; C:extracellular space; C:sarcoplasmic reticulum; C:extracellular exosome; F:integrin binding; F:calcium ion binding; F:heparin binding; P:positive regulation of endothelial cell proliferation; P:negative regulation of angiogenesis; P:regulation of tissue remodeling; P:response to endoplasmic reticulum stress; P:behavioral response to pain; P:positive regulation of peptidyl-tyrosine phosphorylation; P:myoblast migration; P:endothelial cell-cell adhesion; P:positive regulation of neutrophil chemotaxis |
| C078R010 | 2.36 | Ig mu chain C region membrane-bound form | F:protein binding |
| C144R157 | 2.35 | Vitelline membrane outer layer protein 1 homolog precursor | C:extracellular exosome |
| C006R110 | 2.33 | Protein FAM151A | C:extracellular exosome |
| C046R129 | 2.31 | PREDICTED: angiotensin-converting enzyme isoform X2 | C:extracellular space; C:lysosome; C:endosome; C:external side of plasma membrane; C:cell projection part; C:extracellular exosome; C:plasma membrane region; F:actin binding; F:endopeptidase activity; F:carboxypeptidase activity; F:drug binding; F:metallopeptidase activity; F:tripeptidyl-peptidase activity; F:peptidyl-dipeptidase activity; F:zinc ion binding; F:glucosidase activity; F:chloride ion binding; F:mitogen-activated protein kinase kinase binding; F:bradykinin receptor binding; F:mitogen-activated protein kinase binding; P:somitogenesis; P:kidney development; P:blood vessel remodeling; P:angiotensin maturation; P:angiotensin catabolic process in blood; P:regulation of renal output by angiotensin; P:neutrophil mediated immunity; P:antigen processing and presentation of peptide antigen via MHC class I; P:positive regulation of systemic arterial blood pressure; P:cardiac ventricle morphogenesis; P:spermatogenesis; P:determination of left/right symmetry; P:peripheral nervous system development; P:embryonic pattern specification; P:cardioblast differentiation; P:response to organic substance; P:posttranscriptional regulation of gene expression; P:negative regulation of gene expression; P:regulation of vasoconstriction; P:midbrain-hindbrain boundary morphogenesis; P:rhombomere 5 development; P:rhombomere 6 development; P:cerebellum formation; P:locus ceruleus development; P:positive regulation of Wnt signaling pathway; P:positive regulation of cell migration; P:thyroid gland development; P:midbrain development; P:notochord development; P:embryonic camera-type eye development; P:negative regulation of protein binding; P:positive regulation of protein binding; P:mononuclear cell proliferation; P:embryonic heart tube development; P:regulation of vasodilation; P:hormone catabolic process; P:negative regulation of endodermal cell fate specification; P:otic placode formation; P:regulation of bone remodeling; P:determination of dorsal identity; P:paraxial mesoderm development; P:neuron fate specification; P:skeletal system morphogenesis; P:oligodendrocyte differentiation; P:beta-amyloid metabolic process; P:arachidonic acid secretion; P:cartilage development; P:atrial cardiac muscle cell differentiation; P:ventricular cardiac muscle cell differentiation; P:pharyngeal system development; P:heart contraction; P:embryonic retina morphogenesis in camera-type eye; P:hematopoietic stem cell differentiation; P:cilium morphogenesis; P:positive regulation of protein tyrosine kinase activity; P:taste bud formation; P:Kupffer's vesicle development; P:cell proliferation in bone marrow; P:positive regulation of peptidyl-tyrosine autophosphorylation; P:regulation of hematopoietic stem cell proliferation; P:negative regulation of gap junction assembly; P:positive regulation of peptidyl-cysteine S-nitrosylation |
| C255R041 | 2.31 | ---NA--- | - |
| **C075R139** | **2.24** | **Thioredoxin** | **C:extracellular region; C:nucleus; C:mitochondrion; F:protein disulfide oxidoreductase activity; F:oxidoreductase activity, acting on a sulfur group of donors, disulfide as acceptor; P:sulfate assimilation; P:regulation of transcription, DNA-templated; P:protein folding; P:glycerol ether metabolic process; P:response to radiation; P:regulation of protein import into nucleus, translocation; P:cellular response to oxidative stress; P:B cell proliferation; P:positive regulation of DNA binding; P:cell redox homeostasis; P:oxidation-reduction process** |
| C181R143 | 2.22 | PREDICTED: probable polyketide synthase 1 isoform X1 | F:transferase activity; P:metabolic process |
| **C203R158** | **2.19** | **Thioredoxin** | **C:extracellular region; C:nucleus; C:mitochondrion; F:protein disulfide oxidoreductase activity; F:oxidoreductase activity, acting on a sulfur group of donors, disulfide as acceptor; P:sulfate assimilation; P:regulation of transcription, DNA-templated; P:protein folding; P:glycerol ether metabolic process; P:response to radiation; P:regulation of protein import into nucleus, translocation; P:cellular response to oxidative stress; P:B cell proliferation; P:positive regulation of DNA binding; P:cell redox homeostasis; P:oxidation-reduction process** |
| C127R071 | 2.19 | PREDICTED: gastrula zinc finger protein XlCGF57.1-like | F:metal ion binding |
| C034R100 | 2.13 | C-C motif chemokine 8 precursor | C:extracellular region; F:chemokine activity; P:immune response; P:leukocyte chemotaxis; P:regulation of biological process; P:cellular response to cytokine stimulus |
| C006R137 | 2.12 | Chemokine CK-1 | P:positive regulation of leukocyte chemotaxis |
| **C210R115** | **2.11** | **Ependymin precursor** | **C:extracellular region; F:protein C-terminal carboxyl O-methyltransferase activity; F:calcium ion binding; F:C-methyltransferase activity; F:protein-arginine N-methyltransferase activity; F:lysine N-methyltransferase activity; F:tRNA (uracil) methyltransferase activity; F:tRNA (guanine) methyltransferase activity; F:tRNA (cytosine) methyltransferase activity; F:tRNA (adenine-N1-)-methyltransferase activity; F:rRNA (adenine) methyltransferase activity; F:rRNA (cytosine) methyltransferase activity; F:rRNA (uridine) methyltransferase activity; F:Se-methyltransferase activity; P:cell-matrix adhesion** |
| C107R122 | 2.11 | TC1-like transposase | F:nucleotide binding; F:DNA binding; F:transposase activity; P:transposition, DNA-mediated; P:DNA integration |
| C005R140 | 2.10 | 60S acidic ribosomal protein P2 | C:ribosome; F:structural constituent of ribosome; P:translational elongation |
| C160R038 | 2.07 | ---NA--- | - |
| C099R032 | 2.07 | ---NA--- | - |
| **C040R065** | **2.06** | **Thioredoxin** | **C:extracellular region; C:nucleus; C:mitochondrion; F:protein disulfide oxidoreductase activity; F:oxidoreductase activity, acting on a sulfur group of donors, disulfide as acceptor; P:sulfate assimilation; P:regulation of transcription, DNA-templated; P:protein folding; P:glycerol ether metabolic process; P:response to radiation; P:regulation of protein import into nucleus, translocation; P:cellular response to oxidative stress; P:B cell proliferation; P:positive regulation of DNA binding; P:cell redox homeostasis; P:oxidation-reduction process** |
| C113R007 | 2.05 | perilipin-2-like | - |
| C236R012 | 2.04 | PREDICTED: protein NLRC3-like isoform X2 | F:nucleotide binding |
| C125R146 | 2.04 | Mid1-interacting protein 1 | C:nucleus; C:cytosol; C:microtubule; F:protein C-terminus binding; P:negative regulation of microtubule depolymerization; P:positive regulation of fatty acid biosynthetic process; P:protein polymerization; P:positive regulation of ligase activity |
| **C034R078** | **2.03** | **---NA---** | **-** |
| **C110R097** | **2.00** | **Thioredoxin** | **C:extracellular region; C:nucleus; C:mitochondrion; F:protein disulfide oxidoreductase activity; F:oxidoreductase activity, acting on a sulfur group of donors, disulfide as acceptor; P:sulfate assimilation; P:regulation of transcription, DNA-templated; P:protein folding; P:glycerol ether metabolic process; P:response to radiation; P:regulation of protein import into nucleus, translocation; P:cellular response to oxidative stress; P:B cell proliferation; P:positive regulation of DNA binding; P:cell redox homeostasis; P:oxidation-reduction process** |
| C050R107 | 2.00 | Tumor necrosis factor, alpha-induced protein 8-like protein 2 | C:cytoplasm; P:innate immune response; P:negative regulation of inflammatory response; P:negative regulation of T cell activation |
| C189R137 | 1.99 | 60S acidic ribosomal protein P2 | C:ribosome; F:structural constituent of ribosome; P:translational elongation |
| C247R072 | 1.97 | PREDICTED: E3 ubiquitin-protein ligase NEURL3-like | F:zinc ion binding |
| C207R088 | 1.97 | PREDICTED: serine hydroxymethyltransferase, cytosolic | C:nucleoplasm; C:mitochondrion; C:cytosol; C:extracellular exosome; F:glycine hydroxymethyltransferase activity; F:methyltransferase activity; F:pyridoxal phosphate binding; F:protein homodimerization activity; P:glycine metabolic process; P:L-serine catabolic process; P:purine nucleobase biosynthetic process; P:methylation; P:tetrahydrofolate interconversion; P:folic acid metabolic process; P:protein tetramerization |
| C063R163 | 1.96 | ---NA--- | - |
| C255R017 | 1.96 | PREDICTED: indoleamine 2,3-dioxygenase 2-like isoform X1 | F:heme binding; F:dioxygenase activity; P:oxidation-reduction process |
| C211R125 | 1.95 | Thioredoxin | C:extracellular region; C:nucleus; C:mitochondrion; F:protein disulfide oxidoreductase activity; F:oxidoreductase activity, acting on a sulfur group of donors, disulfide as acceptor; P:sulfate assimilation; P:regulation of transcription, DNA-templated; P:protein folding; P:glycerol ether metabolic process; P:response to radiation; P:regulation of protein import into nucleus, translocation; P:cellular response to oxidative stress; P:B cell proliferation; P:positive regulation of DNA binding; P:cell redox homeostasis; P:oxidation-reduction process |
| C101R031 | 1.94 | ---NA--- | - |
| C081R001 | 1.93 | ETS-related transcription factor Elf-3 | C:nucleus; F:RNA polymerase II transcription factor activity, sequence-specific DNA binding; F:sequence-specific DNA binding; P:regulation of transcription from RNA polymerase II promoter; P:cell differentiation |
| C131R026 | 1.92 | 60S acidic ribosomal protein P2 | C:ribosome; F:structural constituent of ribosome; P:translational elongation |
| C137R155 | 1.78 | Tubulin alpha chain-like | C:nucleus; C:cytoplasmic microtubule; C:cytoplasmic ribonucleoprotein granule; C:myelin sheath; C:recycling endosome; C:extracellular exosome; F:double-stranded RNA binding; F:GTPase activity; F:structural constituent of cytoskeleton; F:GTP binding; F:protein domain specific binding; F:ubiquitin protein ligase binding; P:microtubule cytoskeleton organization; P:cytoskeleton-dependent intracellular transport; P:'de novo' posttranslational protein folding; P:protein polymerization; P:cell division; P:cellular response to interleukin-4 |
| C198R059 | 1.75 | Low density lipoprotein receptor | C:lysosome; C:early endosome; C:late endosome; C:Golgi apparatus; C:caveola; C:coated pit; C:external side of plasma membrane; C:integral component of membrane; C:basolateral plasma membrane; C:low-density lipoprotein particle; C:receptor complex; C:apical part of cell; C:recycling endosome membrane; F:glycoprotein binding; F:low-density lipoprotein receptor activity; F:calcium ion binding; F:low-density lipoprotein particle binding; F:very-low-density lipoprotein particle receptor activity; P:response to hypoxia; P:aging; P:cholesterol metabolic process; P:positive regulation of triglyceride biosynthetic process; P:regulation of phosphatidylcholine catabolic process; P:phospholipid transport; P:intestinal cholesterol absorption; P:response to estradiol; P:response to glucagon; P:lipoprotein catabolic process; P:cholesterol import; P:receptor-mediated endocytosis of low-density lipoprotein particle involved in cholesterol transport; P:regulation of cholesterol homeostasis |
| C108R119 | 1.74 | PREDICTED: L-serine dehydratase/L-threonine deaminase | C:mitochondrion; F:L-serine ammonia-lyase activity; F:protein kinase activity; F:L-threonine ammonia-lyase activity; F:ATP binding; F:pyridoxal phosphate binding; F:protein homodimerization activity; P:gluconeogenesis; P:protein phosphorylation; P:L-serine catabolic process; P:threonine catabolic process; P:response to cobalamin; P:pyruvate biosynthetic process; P:response to amino acid |
| C108R054 | 1.70 | Epidermal growth factor receptor pathway substrate 8-like protein 3 | F:protein binding |
| C132R004 | 1.70 | AP-2 complex subunit mu-1 | C:mitochondrion; C:lysosomal membrane; C:cytosol; C:AP-2 adaptor complex; C:transport vesicle; C:secretory granule; C:terminal bouton; C:extracellular exosome; F:signal sequence binding; F:transporter activity; F:lipid binding; F:clathrin adaptor activity; F:low-density lipoprotein particle receptor binding; P:intracellular protein transport; P:synaptic transmission; P:axon guidance; P:viral process; P:Wnt signaling pathway; P:antigen processing and presentation of exogenous peptide antigen via MHC class II; P:negative regulation of epidermal growth factor receptor signaling pathway; P:neurotrophin TRK receptor signaling pathway; P:ephrin receptor signaling pathway; P:regulation of defense response to virus by virus; P:clathrin-mediated endocytosis; P:negative regulation of protein localization to plasma membrane |
| C075R061 | 1.65 | PREDICTED: zinc finger protein Xfin isoform X2 | F:nucleic acid binding; F:metal ion binding; P:regulation of cellular process |
| C263R089 | 1.64 | PREDICTED: ecotropic viral integration site 5 protein homolog isoform X2 | F:GTPase activator activity; P:regulation of GTPase activity |
| C006R091 | 1.48 | B-cell lymphoma 6 protein homolog | C:nucleus; F:RNA polymerase II regulatory region sequence-specific DNA binding; F:transcriptional repressor activity, RNA polymerase II transcription regulatory region sequence-specific binding; F:protein binding; F:metal ion binding; P:negative regulation of transcription from RNA polymerase II promoter; P:type 2 immune response; P:regulation of cell proliferation; P:regulation of cell differentiation; P:regulation of inflammatory response; P:retina morphogenesis in camera-type eye; P:negative regulation of intrinsic apoptotic signaling pathway by p53 class mediator |

^1^Represents the identity of the associated probe on the 4x44K Atlantic salmon microarray. Probes also identified in the SAM analysis are bolded.

^2^Fold change of individual probes as output from RP are reported. Note that fold changes presented in results section of manuscript are from SAM analysis.

^3^Genes were annotated in Blast2GO using the BLASTx algorithm against the non-redundant protein database of NCBI (2015.09.30). The best BLASTx hit that had an expect (E) value <10^-5^ and an informatively named protein product was chosen and is represented.

^4^Gene Ontology (GO) terms mapped to each microarray probe in Blast2GO are indicated. The associated GO category, biological process (P), molecular function (F), and cellular component (C), is also indicated for each term in parentheses.
