# Supplementary material for: Functional Genomic Analysis of the Impact of Camelina (Camelina sativa) Meal on Atlantic Salmon (Salmo salar) Distal Intestine Gene Expression and Physiology
Source: Mar Biotechnol (NY). 2016 Jun 2;18:418–35. doi: 10.1007/s10126-016-9704-x (PMC4911373; doi:10.1007/s10126-016-9704-x)
Supplement: Supplementary file 2 — (DOCX 20 kb) [file 10126_2016_9704_MOESM2_ESM.docx]

Supplemental Table 2. The rank products (RP) method identified 54 features that were significantly down-regulated in the distal intestine of salmon fed the 24CM diet compared to salmon fed control diet (percentage of false-positives 5%).

| **Probe ID^1^** | **Fold change^2^** | **Best named BLASTx hit^3^** | **Gene Ontology^4^** |
| --- | --- | --- | --- |
| C008R016 | -4.19 | Tubulin-specific chaperone A | C:nucleolus; C:cytosol; C:microtubule; C:extracellular exosome; F:tubulin binding; F:poly(A) RNA binding; F:unfolded protein binding; P:tubulin complex assembly; P:post-chaperonin tubulin folding pathway |
| C149R001 | -4.13 | Hexokinase-2 | C:mitochondrial outer membrane; C:cytosol; F:glucokinase activity; F:protein binding; F:ATP binding; F:glucose binding; F:fructokinase activity; F:mannokinase activity; P:cellular glucose homeostasis; P:response to ischemia; P:lactation; P:apoptotic mitochondrial changes; P:negative regulation of mitochondrial membrane permeability; P:regulation of glucose import; P:carbohydrate phosphorylation; P:glucose 6-phosphate metabolic process; P:transmembrane transport; P:canonical glycolysis; P:negative regulation of reactive oxygen species metabolic process |
| C161R081 | -3.88 | ATP-binding cassette sub-family B member 9-like | C:lysosomal membrane; C:early endosome; C:integral component of endoplasmic reticulum membrane; F:ATP binding; F:xenobiotic-transporting ATPase activity; F:oligopeptide-transporting ATPase activity; F:protein homodimerization activity; P:antigen processing and presentation of peptide antigen via MHC class I; P:drug transmembrane transport; P:metabolic process; P:protein transport; P:oligopeptide transmembrane transport; P:xenobiotic transport |
| C074R108 | -3.59 | PREDICTED: isoaspartyl peptidase/L-asparaginase-like | F:hydrolase activity; P:metabolic process |
| C122R113 | -3.39 | PREDICTED: G-protein coupled receptor 39 | C:integral component of membrane; F:G-protein coupled receptor activity; P:G-protein coupled receptor signaling pathway; P:positive regulation of insulin secretion; P:positive regulation of homeostatic process; P:single-multicellular organism process; P:chemical homeostasis |
| C083R085 | -3.28 | Casein kinase 1 gamma 2a | C:cytosol; C:membrane; F:magnesium ion binding; F:glycoprotein binding; F:protein serine/threonine kinase activity; F:ATP binding; F:peptide binding; F:phosphoprotein binding; P:endocytosis; P:regulation of cell shape; P:peptidyl-serine phosphorylation; P:sphingolipid biosynthetic process; P:small molecule metabolic process; P:protein autophosphorylation; P:positive regulation of canonical Wnt signaling pathway |
| C241R120 | -3.25 | Ig kappa chain V region K29-213 | F:protein binding |
| C040R148 | -2.91 | Charged multivesicular body protein 7 | C:ESCRT III complex; F:protein transporter activity; F:hydrolase activity; F:organic cyclic compound binding; F:heterocyclic compound binding; P:organelle organization; P:metabolic process; P:protein transport; P:single-organism cellular process; P:late endosome to vacuole transport |
| C059R116 | -2.91 | PREDICTED: poly(rC)-binding protein 2 isoform X1 | F:RNA binding; F:protein kinase activity; F:ATP binding; P:protein phosphorylation |
| C260R114 | -2.83 | Immunoglobulin light chain precursor | F:protein binding |
| C012R159 | -2.76 | Ig mu chain C region membrane-bound form | F:protein binding |
| C206R076 | -2.70 | Ig kappa chain V-III region CLL precursor | F:protein binding |
| C221R049 | -2.63 | PREDICTED: zinc finger protein 883-like | F:binding |
| C122R026 | -2.56 | Ig mu chain C region membrane-bound form | F:protein binding; P:response to lipopolysaccharide |
| C139R130 | -2.52 | PREDICTED: E3 ubiquitin-protein ligase RNF213 | F:nucleotide binding; F:ubiquitin-protein transferase activity; F:ATPase activity; F:metal ion binding; P:sprouting angiogenesis |
| C173R106 | -2.51 | Ig mu chain C region membrane-bound form | F:protein binding |
| C144R034 | -2.51 | DNA replication complex GINS protein PSF3 | C:GINS complex; F:chromatin binding; P:mitotic S phase; P:mitotic cell cycle; P:DNA strand elongation involved in DNA replication; P:heart development |
| C116R107 | -2.48 | Immunoglobulin light chain precursor | F:protein binding |
| C246R064 | -2.44 | Immunoglobulin delta heavy chain constant region, partial | C:integral component of membrane; F:protein binding |
| C061R142 | -2.42 | Immunoglobulin delta heavy chain constant region, partial | C:integral component of membrane; F:protein binding |
| C168R074 | -2.39 | PREDICTED: nocturnin | F:molecular_function; P:biological_process; C:cellular_component |
| C264R117 | -2.36 | Ig mu chain C region membrane-bound form | F:protein binding |
| C147R144 | -2.30 | Epididymal secretory protein E1 precursor | C:lysosome; C:endoplasmic reticulum; C:extracellular exosome; F:cholesterol binding; F:enzyme binding; P:cholesterol metabolic process; P:response to virus; P:intracellular cholesterol transport; P:cholesterol efflux; P:cholesterol homeostasis |
| C151R089 | -2.29 | Claudin-3 | C:bicellular tight junction; C:integral component of membrane; C:lateral plasma membrane; C:extracellular exosome; F:structural molecule activity; F:identical protein binding; P:response to hypoxia; P:epithelial cell morphogenesis; P:calcium-independent cell-cell adhesion via plasma membrane cell-adhesion molecules |
| C125R032 | -2.24 | Immunoglobulin mu heavy chain, partial | F:protein binding |
| C183R035 | -2.23 | Receptor-transporting protein 3 | F:molecular_function; P:biological_process; C:cellular_component; C:cytoplasm; P:protein targeting to membrane; P:detection of chemical stimulus involved in sensory perception of bitter taste |
| C102R039 | -2.22 | PREDICTED: barrier-to-autointegration factor-like | C:nucleoplasm; C:chromosome; C:cytosol; C:extracellular exosome; F:DNA binding; F:protein binding; P:mitotic prophase; P:mitotic anaphase; P:mitotic nuclear envelope disassembly; P:mitotic nuclear envelope reassembly; P:response to virus; P:DNA integration; P:establishment of integrated proviral latency |
| C123R027 | -2.18 | ---NA--- | - |
| C164R003 | -2.15 | Proteasome subunit beta type 8 | C:nucleus; C:cytoplasm; C:proteasome core complex; C:extracellular exosome; C:spermatoproteasome complex; F:threonine-type endopeptidase activity; P:proteasomal ubiquitin-independent protein catabolic process; P:antigen processing and presentation; P:proteasome-mediated ubiquitin-dependent protein catabolic process; P:fat cell differentiation |
| C002R100 | -2.14 | pol-like protein | P:RNA-dependent DNA replication; F:RNA-directed DNA polymerase activity; F:metal ion binding; F:nucleic acid binding; F:zinc ion binding |
| C028R010 | -2.14 | NADH dehydrogenase 1 alpha subcomplex subunit 2 | C:mitochondrial respiratory chain complex I; F:oxidoreductase activity; P:transport; P:oxidation-reduction process |
| C092R031 | -2.12 | Immunoglobulin delta heavy chain membrane-bound form | F:protein binding |
| C153R027 | -2.10 | PREDICTED: olfactomedin-4-like | P:positive regulation of substrate adhesion-dependent cell spreading; P:protein homooligomerization; F:protein homodimerization activity; C:perinuclear region of cytoplasm; C:extracellular exosome; P:negative regulation of I-kappaB kinase/NF-kappaB signaling; P:negative regulation of immune response; C:plasma membrane; C:specific granule; F:cadherin binding; C:extracellular space; F:catalytic activity; P:metabolic process |
| C243R088 | -2.10 | Immunoglobulin light chain | F:protein binding |
| C215R037 | -2.08 | PREDICTED: olfactomedin-4-like | C:cell part; P:regulation of biological process |
| C143R043 | -2.03 | PREDICTED: retinoic acid receptor RXR-gamma-B-like isoform X1 | C:nucleus; F:steroid hormone receptor activity; F:9-cis retinoic acid receptor activity; F:zinc ion binding; F:sequence-specific DNA binding; P:steroid hormone mediated signaling pathway; P:positive regulation of transcription from RNA polymerase II promoter |
| C236R100 | -2.02 | Immunoglobulin light chain precursor | F:protein binding |
| C259R141 | -2.01 | 40S ribosomal protein S2 | C:nucleoplasm; C:nucleolus; C:focal adhesion; C:membrane; C:cytosolic small ribosomal subunit; C:extracellular exosome; F:mRNA binding; F:structural constituent of ribosome; F:fibroblast growth factor binding; F:enzyme binding; F:protein complex binding; P:ribosomal small subunit assembly; P:nuclear-transcribed mRNA catabolic process, nonsense-mediated decay; P:formation of translation preinitiation complex; P:translational elongation; P:translational termination; P:SRP-dependent cotranslational protein targeting to membrane; P:acute-phase response; P:viral transcription; P:positive regulation of transferase activity; P:cellular response to interleukin-4 |
| C021R036 | -1.99 | Type I keratin E7 | C:intermediate filament; F:structural molecule activity; P:cell migration involved in gastrulation |
| C166R162 | -1.99 | Immunoglobulin light chain precursor | - |
| C205R051 | -1.98 | Ig mu chain C region membrane-bound form | F:protein binding |
| C022R068 | -1.94 | Type-2 ice-structuring protein precursor | C:integral component of plasma membrane; F:purinergic nucleotide receptor activity; F:extracellular ATP-gated cation channel activity; F:ATP binding; P:response to ATP; P:purinergic nucleotide receptor signaling pathway; P:cation transmembrane transport |
| C108R157 | -1.88 | Ig kappa chain V-IV region B17 precursor | F:protein binding |
| C024R006 | -1.85 | Ig kappa chain V-III region CLL precursor | F:protein binding |
| C082R022 | -1.83 | Lymphocyte G0/G1 switch protein 2 | C:mitochondrion; P:positive regulation of extrinsic apoptotic signaling pathway |
| C249R068 | -1.83 | Immunoglobulin light chain precursor | F:protein binding |
| C108R121 | -1.82 | PREDICTED: small ubiquitin-related modifier 1-B-like | F:protein binding |
| C236R044 | -1.81 | PREDICTED: ribosome-releasing factor 2, mitochondrial | C:mitochondrial matrix; F:translation elongation factor activity; F:GTPase activity; F:protein binding; F:GTP binding; P:ribosome disassembly; P:mitochondrial translational elongation; P:mitochondrial translational termination |
| C166R163 | -1.70 | Leukocyte elastase inhibitor | C:extracellular space; F:serine-type endopeptidase inhibitor activity; P:negative regulation of endopeptidase activity |
| C130R113 | -1.69 | Ras-related protein Rap-1b precursor | C:lipid particle; C:cytosol; C:plasma membrane; C:cell-cell junction; C:membrane raft; C:extracellular exosome; F:GTPase activity; F:GTP binding; F:GDP binding; F:protein complex binding; P:intracellular protein transport; P:nucleocytoplasmic transport; P:heart development; P:cell proliferation; P:response to carbohydrate; P:microvillus assembly; P:platelet activation; P:Rap protein signal transduction; P:somite rostral/caudal axis specification; P:cellular response to drug; P:negative regulation of calcium ion-dependent exocytosis; P:convergent extension; P:establishment of endothelial barrier; P:positive regulation of ERK1 and ERK2 cascade; P:cellular response to cAMP; P:cellular response to gonadotropin-releasing hormone; P:regulation of cell junction assembly; P:regulation of establishment of cell polarity; P:negative regulation of synaptic vesicle exocytosis |
| C044R082 | -1.66 | Mitochondrial 2-oxoglutarate/malate carrier protein | C:nucleus; C:mitochondrial inner membrane; C:integral component of membrane; F:transporter activity; F:poly(A) RNA binding; P:mitochondrial transport |
| C188R129 | -1.54 | Ig kappa chain V region K29-213 | F:protein binding |
| C105R067 | -1.31 | ---NA--- | - |
| C183R024 | -1.13 | PREDICTED: vascular endothelial growth factor receptor 1 | - |

^1^Represents the identity of the associated probe on the 4x44K Atlantic salmon microarray.

^2^Fold change of individual probes as output from RP are reported. Note that fold changes presented in results section of manuscript are from SAM analysis.

^3^Genes were annotated in Blast2GO using the BLASTx algorithm against the non-redundant protein database of NCBI (2015.09.30). The best BLASTx hit that had an expect (E) value <10^-5^ and an informatively named protein product was chosen and is represented.

^4^Gene Ontology (GO) terms mapped to each microarray probe in Blast2GO are indicated. The associated GO category, biological process (P), molecular function (F), and cellular component (C), is also indicated for each term in parentheses.
